# Gallylene-Mediated C–C Bond Activation of Diphenylcyclopropenone and Chalcogen Atom Insertion into a Ga–C Bond

**DOI:** 10.1021/acsorginorgau.6c00053

**Published:** 2026-07-14

**Authors:** Nijito Mukai, Sota Iwasaki, Takuya Kodama, Mamoru Tobisu

**Affiliations:** ∇ Department of Applied Chemistry, Graduate School of Engineering, 13013 The University of Osaka, Suita, Osaka 565-0871, Japan; ‡ Innovative Catalysis Science Division, Institute for Open and Transdisciplinary Research Initiatives (ICS-OTRI), 13013 The University of Osaka, Suita, Osaka 565-0871, Japan; § Center for Future Innovation, Graduate School of Engineering, 13013 The University of Osaka, Suita, Osaka 565-0871, Japan

**Keywords:** gallium, cyclopropenone, oxidative addition, C−C bond activation, insertion

## Abstract

We report the synthesis and structural characterization of a gallacyclobutenone. The gallacyclobutenone was synthesized via C–C bond oxidative addition of cyclopropenone to a Ga­(I) species bearing a phenalenyl-based bidentate ligand. Density functional theory (DFT) calculations suggest that the oxidative addition proceeds through nucleophilic attack of the gallylene on the alkene moiety of the cyclopropenone, ring opening with C–C bond cleavage to generate a ketene intermediate, and subsequent cyclization through Ga–C bond formation. The resulting four-membered gallacycle undergoes oxygen- and sulfur-atom insertion reactions into the Ga–C­(=O) bond, affording the corresponding five-membered gallacycles.

## Graphical abstract

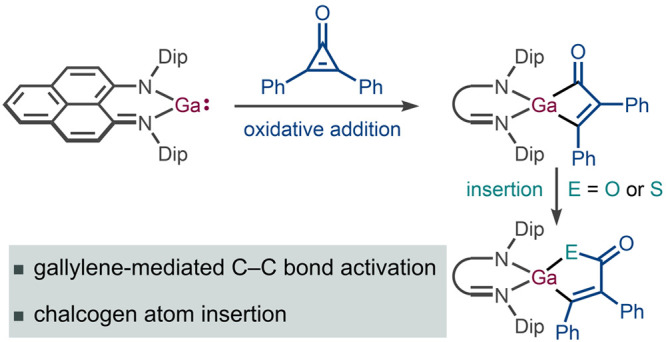

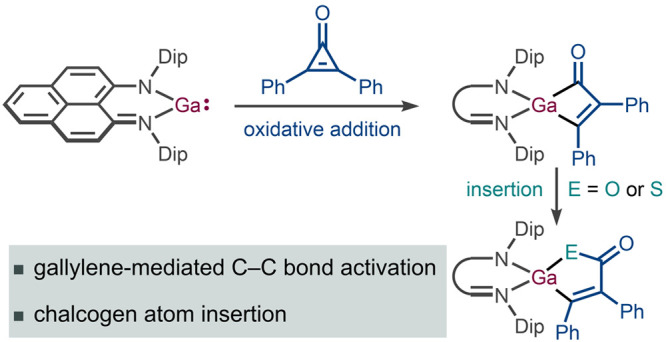

Group 13 element compounds, with the exception of those of thallium, predominantly feature the +3 oxidation state and have been studied as Lewis acidic species in areas such as catalysis and materials science based on their electron-deficient nature.^1^ By contrast, group 13 compounds in the neutral +1 oxidation state exhibit an ns^2^ electron configuration and are regarded as group 13 metallylenes.^2–5^ Featuring a lone pair and a low-lying vacant orbital, these species display ambiphilic character. Recent advances in ligand design have enabled the isolation of these low valent species, which exhibit unusual electronic structures and intriguing chemical behavior. This ambiphilic character, arising from the combination of a lone pair and a vacant p orbital, enables cooperative substrate activation.^3,4^ As a consequence, low-valent group 13 species can undergo oxidative addition reactions accompanied by oxidation-state changes from E­(I) to E­(III), a reactivity pattern fundamentally distinct from that of conventional trivalent group 13 compounds and in some cases reminiscent of transition-metal complexes.

To date, oxidative addition involving a variety of σ-bonds at group 13 elements has been reported; however, the cleavage of C–C bonds remains particularly challenging.^6^ In recent years, several examples of C–C bond oxidative addition at borylenes and alumylenes have been disclosed (Figure 1).^7^ In 1996, Power and co-workers reported that a borylene generated *in situ* undergoes intramolecular oxidative addition of a C­(aryl)–Me bond to afford a cyclic boron­(III) compound.^8^ More recently, remarkable progress has been achieved in the oxidative addition of C–C bonds by neutral and anionic Al­(I) complexes. In 2019, Aldridge reported the reversible oxidative addition of a benzene C–C bond via a two-step mechanism involving a norcaradiene-type intermediate.^9^ Crimmin demonstrated that an alumylene supported by a β-diketiminate ligand undergoes oxidative addition of the C–C bond of methylidene cycloalkane derivatives.^10^ Kinjo reported oxidative addition of the C–C bond of the four-membered ring in biphenylene by an anionic aluminum­(I) complex, whereas Liu reported oxidative addition of the aromatic C–C bond in biphenylene by a neutral aluminum­(I) complex bearing a carbazole-type ligand.^11,12^ In contrast, analogous reactions involving heavier group 13 metallylenes remain rare, primarily because the reactivity of Ga­(I) and In­(I) species is generally diminished relative to that of Al­(I) and B­(I) owing to the inert-pair effect.^1a^ Indeed, to the best of our knowledge, the only reported example is the oxidative addition of a C­(aryl)–C­(alkyl) bond adjacent to an azo group by a Ga­(I) species, as reported by Power.^13^ Consequently, the development of C–C bond activation at heavier low-valent group 13 centers remains largely unexplored.

**Figure 1:**
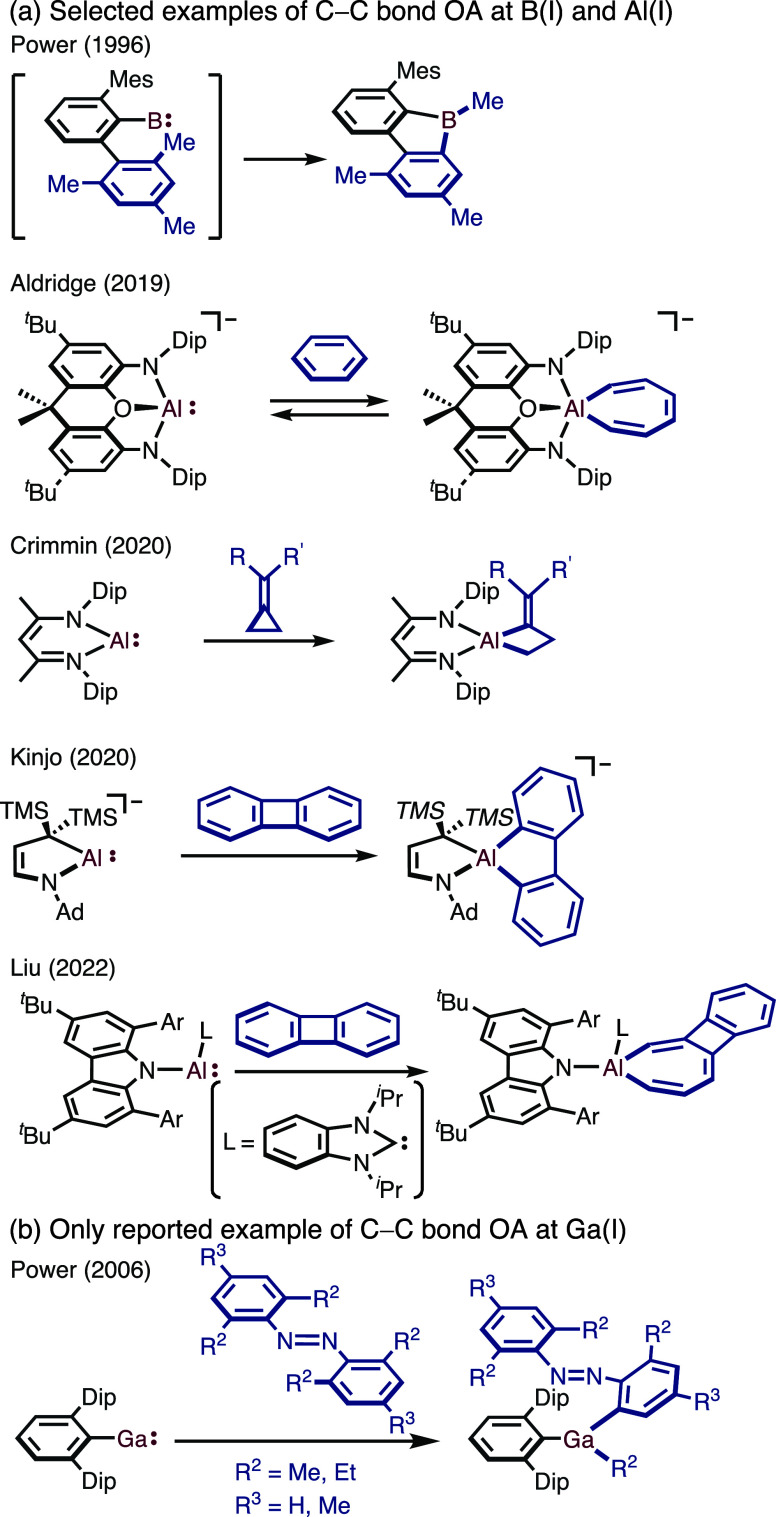
Overview of C–C bonds oxidative addition (OA) at group 13 elements. Mes = mesityl, Dip = 2,6-diisopropylphenyl, TMS = trimethylsilyl, Ar = 3,5-di-*tert*-butylphenyl.

Within this context, strained carbocyclic systems represent promising substrates for bond activation chemistry because ring strain facilitates C–C bond cleavage, while the resulting metallacycles often retain substantial strain energy that can be exploited in subsequent transformations.^14,15^

Herein, we report the oxidative addition of the C–C bond of diphenylcyclopropenone to gallylene **1** ^16^ bearing a phenalenyl-based bidentate ligand,^17^ as well as the subsequent reactivity of the resulting complexes **2**.

Treatment of gallylene **1** with diphenylcyclopropenone at room temperature for 16 h afforded the four-membered gallacycle **2** in 35% isolated yield (Scheme 1). Single crystals suitable for X-ray diffraction analysis were obtained by recrystallization from hexane (Figure 2). The solid-state molecular structure revealed that the four-membered ring of **2** adopts a distorted geometry with an acute C14–Ga1–C16 angle of 69.6 (1)°. In addition, the C14–O1 bond length was determined to be 1.196 (4) Å, indicating substantial double-bond character. This observation is consistent with the ^13^C­{^1^H} NMR spectrum, in which the resonance attributable to the carbonyl carbon appears at 209.7 ppm. Four-membered metallacyclobutenone frameworks have also been reported for aluminum systems, in which insertion of carbon monoxide into an aluminacyclopropene, generated from the reaction of an Al­(I) species with an alkyne, affords an aluminacyclobutenone.^18^ In contrast, extension of this strategy to gallium remains challenging, likely because the larger atomic radius of gallium disfavors the formation of highly strained gallacyclopropene intermediates. The present results therefore demonstrate that gallacyclobutenones can instead be accessed through a distinct synthetic strategy based on C–C bond oxidative addition. Furthermore, the reactivity of gallylene **1** toward cyclobutenone was also investigated; however, no oxidative addition was observed, and **1** as quantitatively recovered.

**Scheme 1:**
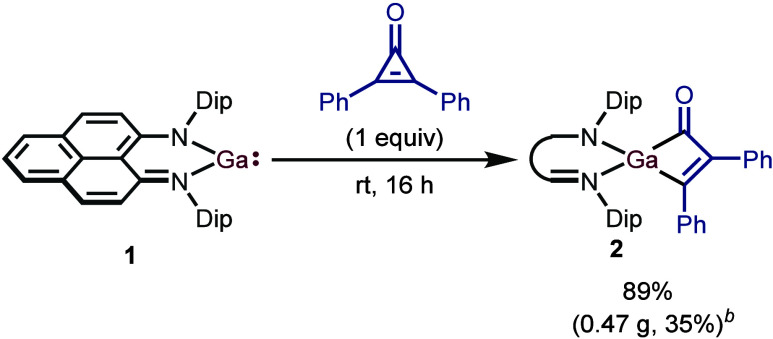
Reaction of Diphenylcyclopropenone with **1** ^a^

a Reaction conditions: **1** and diphenylcyclopropenone (1 equiv) in benzene-*d*_6_ at rt for 16 h. Yield was determined by ^1^H NMR using 1,3,5-trimethoxybenzene (TMB) as an internal standard.
b Run using **1** (1.0 g, 1.7 mmol) and diphenylcyclopropenone (0.71 g, 3.4 mmol) in toluene. Isolated yields are shown.

**Figure 2:**
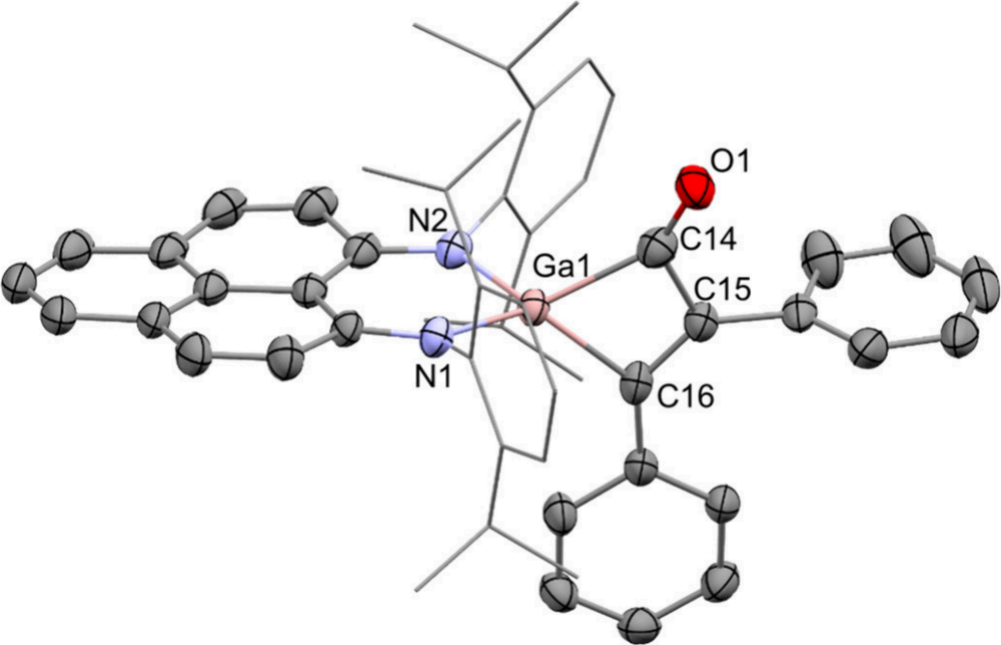
ORTEP drawing of **2** depicted at the 50% probability level. Hydrogen atoms are omitted for clarity. Aryl groups are depicted as wireframe forms. Selected bond distances (Å), bond angles (deg) for **2**: Ga1–N1, 1.921 (2); Ga1–N2, 1.944 (2); Ga1–C14, 2.069 (3); Ga1–C16, 2.010 (3); C14–O1, 1.196 (4); C14−C15, 1.512 (4); C15–C16, 1.355 (4); N1–Ga1–N2, 94.1 (1); C14–Ga1–C16, 69.6 (1); Ga1–C14–C15, 87.3 (2); C14–C15–C16, 108.4 (2); C15–C16–Ga1, 94.1 (2).

The reaction mechanism was investigated by density functional theory (DFT) calculations at the PBE0/def2-SVP level of theory (Figure 3).^19^ The calculated reaction profile indicates that the oxidative addition is initiated by nucleophilic attack of **1** on the alkene moiety of diphenylcyclopropenone via **TS1**, with a relatively low activation barrier of Δ*G* ^‡^ = 9.5 kcal/mol. This step is followed by ring opening via C–C bond cleavage to afford **INT2** bearing a ketene moiety.^20^ The Ga–C bond in **INT2** features a short bond length of 1.852 Å, which is approximately 0.14 Å shorter than reported Ga–Me single bonds.^21,22^ Subsequent intramolecular Ga–C bond formation between the central carbon atom of the ketene fragment and the gallium center proceeds through **TS2** with a small activation barrier of Δ*G* ^‡^ = 5.6 kcal/mol, affording **2**. Overall, the computed mechanism parallels that proposed by Cummins and co-workers for the reaction of phosphinidenes [P­(I)] with cyclopropenone derivatives, suggesting that analogous reaction pathways may also be accessible to low-valent group 13 species.^23^

**Figure 3:**
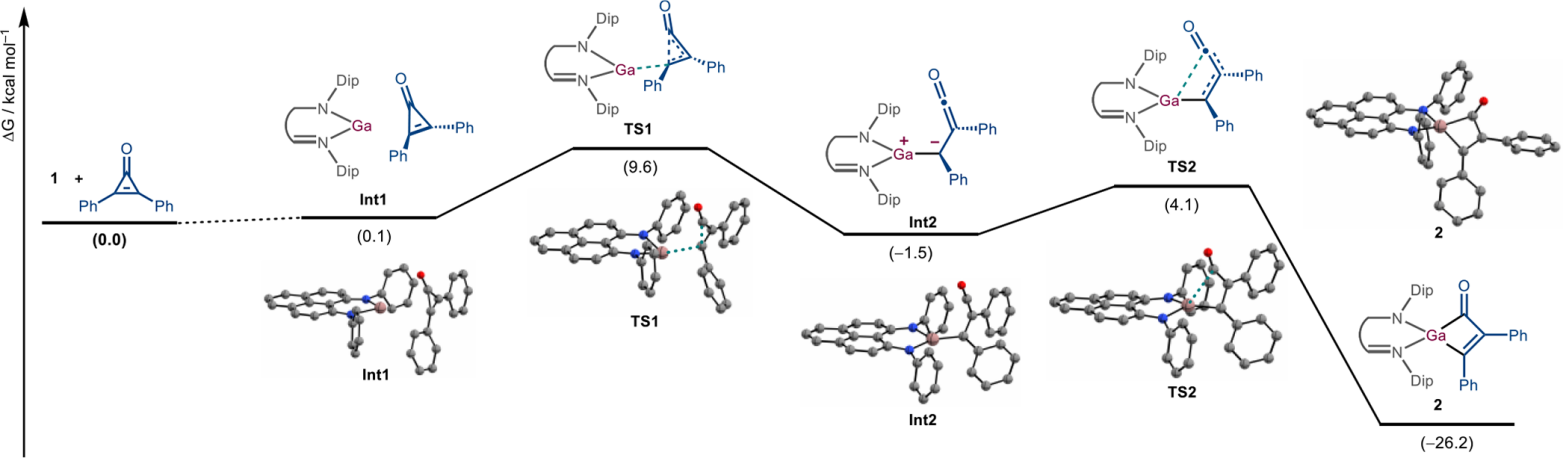
DFT-computed energy profile for the oxidative addition of diphenylcyclopropenone into **1** at the PBE0-D3­(BJ)/def2-SVP level of theory. Isopropyl groups are omitted for clarity.

Next, we turned our attention to the reactivity of the strained four-membered gallacycle **2** (Scheme 2). In particular, insertion reactions are synthetically attractive because they enable simultaneous modification of both the ring size and elemental composition in a single step.^24,25^ Although insertion reactions with isocyanides and various π-components, including alkenes and alkynes, were investigated, no reaction occurred and **2** was recovered unchanged. In contrast, oxygen-atom insertion proceeded smoothly. Specifically, exposure of **2** to an oxygen atmosphere at room temperature for 20 h resulted in oxygen-atom insertion into a Ga–C­(=O) bond, affording oxygen-inserted product **3** in 51% isolated yield. Related reactivity has previously been reported by Cui and co-workers, who demonstrated that an aluminacyclopropenone bearing a β-diketiminate ligand undergoes oxygen-atom insertion under an oxygen atmosphere at – 20 °C to furnish the corresponding five-membered aluminacycle.^18^

**Scheme 2:**
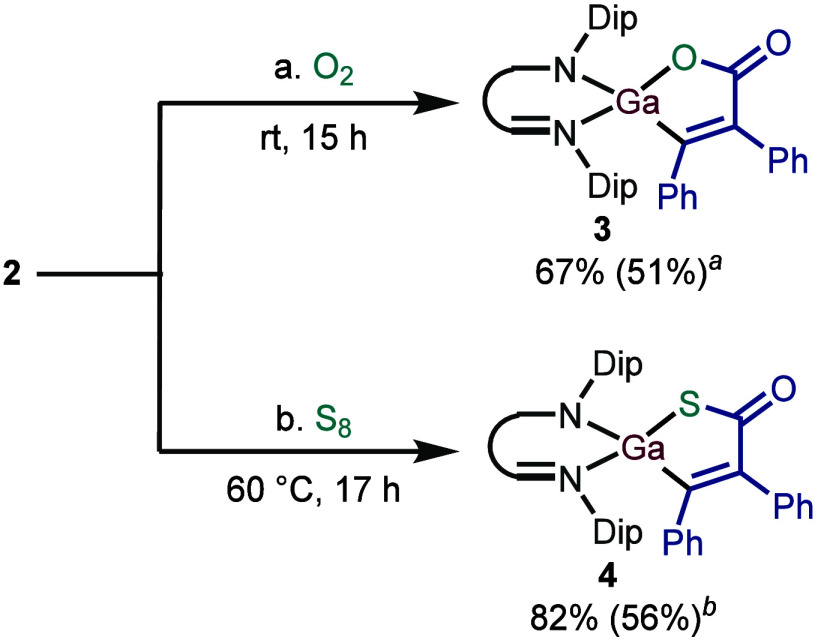
Reaction of **2** with O_2_ and S_8_ [Fn sch2-fn3]

† Reaction conditions: (a) **2** under O_2_ (1 atm) in benzene-*d*_6_ at rt for 15 h. Yield was determined by ^1^H NMR using TMB as an internal standard. (b) **2** and S_8_ (0.31 equiv) in benzene-*d*_6_ at 60 °C for 17 h. Yield was determined by ^1^H NMR using TMB as an internal standard. Isolated yields are shown.
a Run in toluene for 20 h. Isolated yield is shown.
b
**1** and diphenylcyclopropenone (1 equiv) in toluene at 60 °C for 0.25 h; S_8_ (0.13 equiv) at 60 °C for 16 h.

Sulfur atom insertion was also investigated. After optimization of the reaction conditions, treatment of **2** with S_8_ at 60 °C for 16 h afforded the corresponding sulfur-inserted product **4** in 82% yield. To the best of our knowledge, sulfur atom insertion into a gallacyclic framework has not previously been reported. Notably, both the oxygen- and sulfur-insertion reactions proceeded with complete selectivity for monoatom insertion, and no products containing multiple inserted heteroatoms were detected.^26^

The ring-expanded insertion products were unambiguously characterized by single-crystal X-ray diffraction analysis (Figure 4). The C38–O1 bond lengths in the solid state were determined to be 1.220 (3) Å for **3** and 1.213 (5) Å for **4**, indicating that the carbonyl bond in **3** is slightly elongated relative to that in **4**. This observation is consistent with the ^13^C­{^1^H} NMR spectra measured in solution, in which the carbonyl resonance of **3** appeared at 171.9 ppm, whereas that of **4** was observed further downfield at 202.3 ppm. These results suggest a greater degree of electron donation from the inserted oxygen atom into the carbonyl π* orbital in **3**, resulting in greater charge delocalization and a more pronounced ionic character relative to that in **4**. Consistent with this interpretation, the calculated Wiberg bond index (WBI) for the Ga1–O2 bond in **3** (0.371) is substantially smaller than that for Ga1–S1 bond in **4** (0.781).^27^ Such electronic delocalization also influences the coordination geometry around the gallium center. Specifically, the sum of the N1–Ga1–N2, N1–Ga1–C40, and C40–Ga1–N2 bond angles in **3** is 350.2°, indicating a highly planar coordination environment around gallium. In contrast, the corresponding sum of bond angles in **4** is significantly smaller (335.2°), reflecting a substantially more pyramidal geometry.

**Figure 4:**
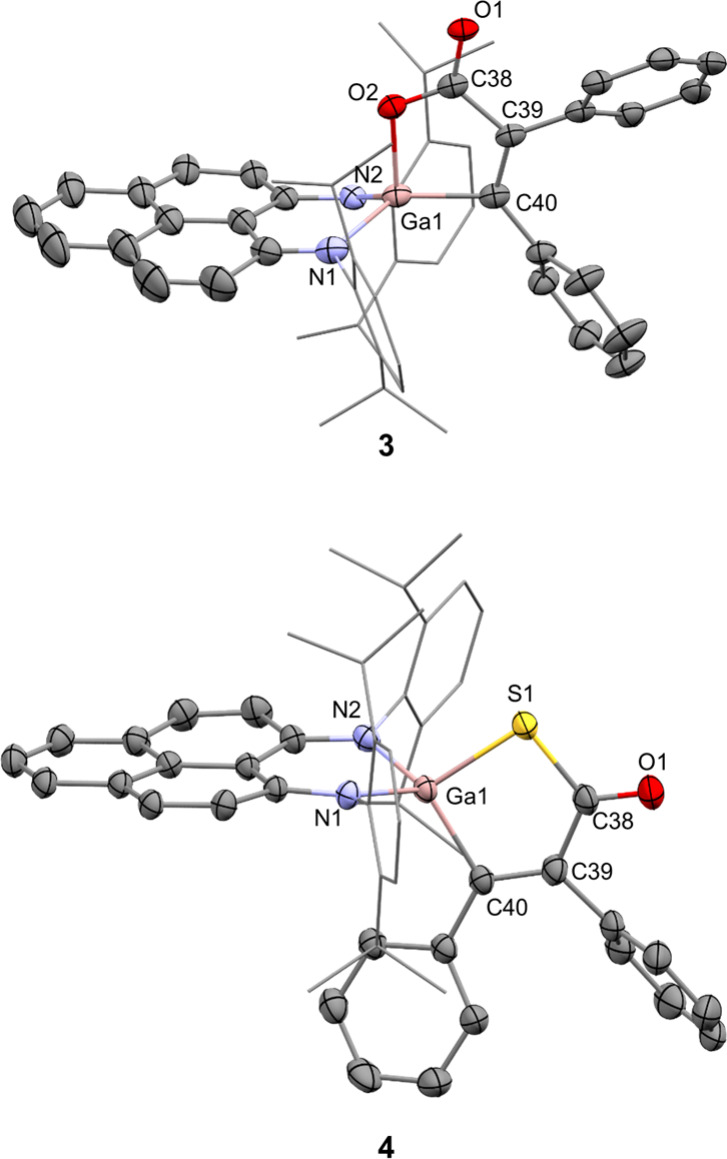
ORTEP drawings of **3** (top) and **4** (bottom) shown with thermal ellipsoids at the 30% (**3**) and 50% (**4**) probability levels, respectively. Hydrogen atoms are omitted for clarity. Dip groups are depicted as wireframe forms. Selected bond distances (Å), bond angles (deg) for **3**: Ga1–N1, 1.908 (2); Ga1–N2, 1.890 (2); Ga1–O2, 1.900 (1); Ga1–C40, 1.961 (2); C38–O1, 1.220 (3); C38–O2, 1.310 (3); C38–C39, 1.519 (3); C39–C40, 1.348 (3); N1–Ga1–N2, 95.57 (7); O2–Ga1–C40, 89.22(8); Ga1–O2–C38, 110.9 (1); O2–C38–C39, 115.8 (2); C38–C39–C40, 117.5 (2); C39–C40–Ga1, 106.2 (2); N1–Ga1–C40, 124.95(9); C40–Ga1–N2, 129.70(8). Selected bond distances (Å), bond angles (deg.) for **4**: Ga1–N1, 1.925 (3); Ga1–N2, 1.933 (3); Ga1–S1, 2.247 (1); Ga1–C40, 1.993 (3); C38–O1, 1.213 (5); C38–S1, 1.791 (5); C38–C39, 1.504 (6); C39–C40, 1.504 (6); N1–Ga1–N2, 94.8 (1); S1–Ga1–C40, 94.5 (1); Ga1–S1–C38, 92.6 (1); S1–C38–C39, 119.4 (3); C38–C39–C40, 121.5 (3); C39–C40–Ga1, 118.3 (3); N1–Ga1–C40, 119.7 (1); C40–Ga1–N2, 120.7 (1).

In summary, we demonstrated that gallylene **1** reacts with diphenylcyclopropenone to afford the C–C bond oxidative addition complex **2**. The molecular structure of **2** was unequivocally determined by single-crystal X-ray diffraction analysis, revealing a distorted four-membered gallacyclic framework. This reaction represents the first example of a gallacyclobutenone and provides access to a structural motif that cannot be readily synthesized using methodologies developed for analogous aluminum systems. DFT calculations indicated that the reaction proceeds through nucleophilic attack of **1** on the alkene moiety of the cyclopropenone, generating **INT2** featuring a ketene moiety, followed by intramolecular Ga–C bond formation to afford **2**. Furthermore, **2** undergoes ring-expansion reactions via insertion of oxygen and sulfur atoms, and the molecular structures of the resulting products were also established by single-crystal X-ray diffraction analysis.

## Supplementary Material



## Data Availability

The data underlying this study are available in the published article and its Supporting Information.

## Notes

Detailed experimental procedures, characterization of new compounds, crystallographic data for compounds **2**–**4**, NMR spectra, HRMS spectra, and computational details (PDF)
