# Supplementary material for: Gallylene-Mediated C–C Bond Activation of Diphenylcyclopropenone and Chalcogen Atom Insertion into a Ga–C Bond
Source: ACS Org Inorg Au. 2026 Jul 14;6(4):392–7. doi: 10.1021/acsorginorgau.6c00053 (PMC13449436; doi:10.1021/acsorginorgau.6c00053)
Supplement: Supplementary file 1 [file gg6c00053_si_001.pdf]

Supporting Information

For

Gallylene-Mediated C–C Bond Activation of Diphenylcyclopropenone and Chalcogen Atom Insertion into a Ga–C Bond

Nijito Mukai,† Sota Iwasaki,† Takuya Kodama,*,†,‡,§ and Mamoru Tobisu*,†,‡

†*Graduate School of Engineering, The University of Osaka, Japan.*

‡*ICS-OTRI, The University of Osaka, Japan.*

§*Center for Future Innovation, Graduate School of Engineering, The University of Osaka, Japan.*

kodama@chem.eng.osaka-u.ac.jp
tobisu@chem.eng.osaka-u.ac.jp

CONTENTS

I. General Information S2

II. Materials S2

III. Oxidative Addition of a C–C Bond into 1 and Chalcogen Atom Insertion into a Ga–C Bond S3

IV. Synthesis of Gallium Complexes S6

V. Trapping Experiment of Ketene Intermediate INT2 .... S15

VI. Crystallographic Information S16

VII. References S17

VIII. Cartesian Coordinates of Optimized Structure S18

I. General Information

^{1}H and ^{13}C NMR spectra were recorded on JEOL ECS-400 spectrometers in $CDCl_3$, benzene-d_6. The chemical shifts in ^{1}H NMR spectra were determined relative to $CHCl_3$ (δ 7.26), C_6H_6 (δ 7.16), toluene (δ 2.08). The chemical shifts in $^{13}C\{^1H\}$ NMR spectra were recorded relative to $CHCl_3$ (δ 77.16). The data are reported as follows: chemical shift (δ) in ppm, coupling constant (Hz), and integration. NMR yields were determined using 1,3,5-trimethoxybenzene (TMB) as internal standards. Infrared (IR) spectra was obtained using a JASCO FT/IR-4200 spectrometer. Absorption is reported in reciprocal centimeters (cm^{-1}). High resolution mass spectra (HRMS) were obtained using a JMS-T100LP (DART-TOF) spectrometer. Melting points were determined using a Yamato melting point apparatus. X-ray crystal analyses were performed on a Rigaku/XtaLABPro P200 Hybrid Photon Counting diffractometer (Cu-Kα, λ = 1.54184 Å). The structures were solved with olex2.solve 1.5 and refined with SHELXL−2018/3). All calculations were conducted using the Gaussian 09 suite program (G09 Rev C.01)[1] at the PBE0-D3(BJ)/def2-TZVPP//PBE0-D3(BJ)/ def2-SVP level. Electronic structures and properties were analyzed by the Natural Bond Orbitals (NBO)[2] version 3.1[3] method.

II. Materials

All commercially available reagents and solvents were supplied from TCI, WAKO and Aldrich. All reactions with moisture- or air-sensitive compounds were performed in a nitrogen-filled glove box. **1**[CAS 2922679-05-6] was prepared according to a literature procedure.[4] 2-Isocyano-1,3-dimethylbenzene[2769-71-3] was prepared according to a literature procedure.[5]

III. Oxidative Addition of a C–C Bond into 1 and Chalcogen Atom Insertion into a Ga–C Bond

General procedure for NMR experiments

(i) Reaction of 1 with diphenylcyclopropenone

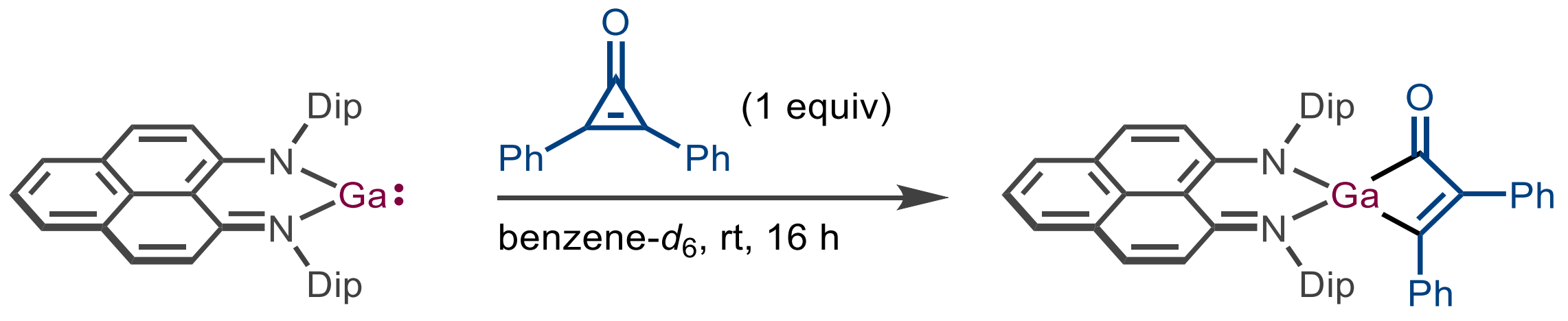

1 **2**

A benzene-d_6 (0.5 mL) solution of **1** (18 mg, 0.031 mmol), diphenylcyclopropenone (6.3 mg, 0.031 mmol) was transferred into a J. Young NMR tube. The tube was then sealed with screwcap, and the mixture was reacted at rt for 16 h under dark. After the reaction, 1,3,5-trimethoxybenzene (TMB) was added to tube under dark as an internal standard. The yield of **2** was determined by the *in situ* comparison of the new resonance at 3.58 ppm (2H) in the ^{1}H NMR spectrum to the TMB (89%).

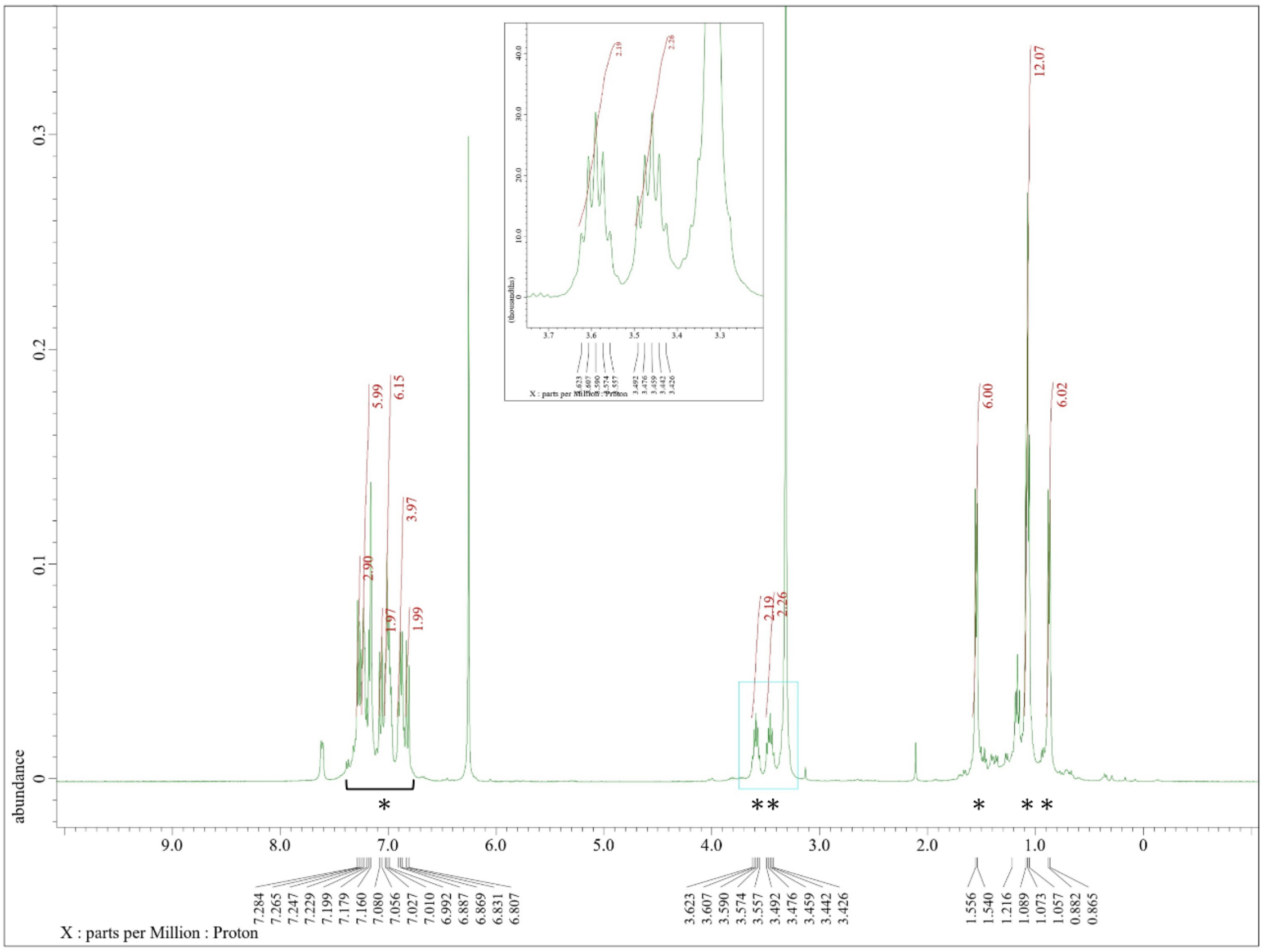

Figure S1. Crude ^{1}H NMR spectra (400 MHz) of the conversion of **1** to **2** in benzene-d_6. Asterisks (*) indicate signals assigned to **2**.

(ii) Oxygen atom insertion into 2

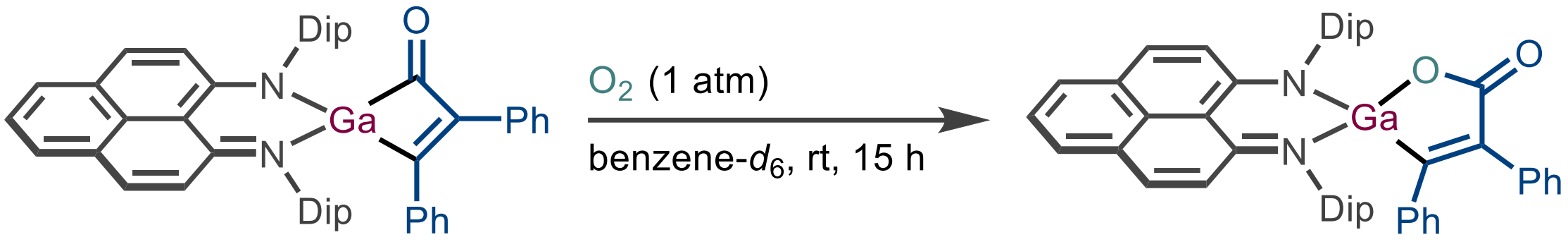

In a nitrogen-filled glovebox, a solution of **2** (18 mg, 0.020 mmol) benzene-d_6 (0.5 mL), and the mixture was degassed by freeze pomp saw in three times. Then, O_2 (1 atm) was transferred into the tube, and the mixture was stirred at rt for 15 h. After the reaction, 1,3,5-trimethoxybenzene (TMB) was added to tube under dark as an internal standard. The yield of **3** was determined by the *in situ* comparison of the new resonance at 3.88 ppm (2H) in the ^{1}H NMR spectrum to the TMB (67%).

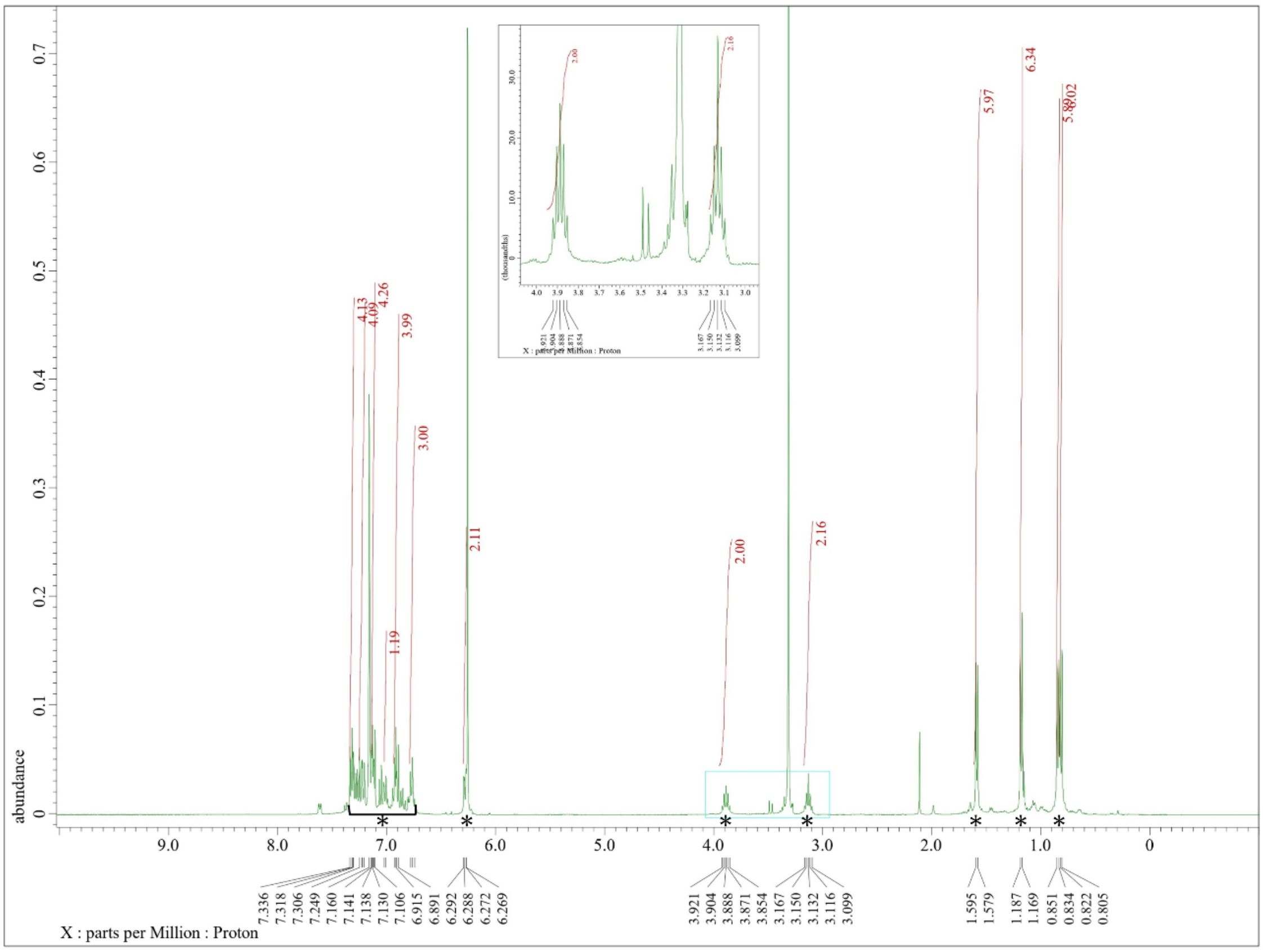

Figure S2. Crude ^{1}H NMR spectra (400 MHz) of the conversion of **2** to **3** in benzene-d_6. Asterisks (*) indicate signals assigned to **3**.

(iii) Sulfur atom insertion into 2

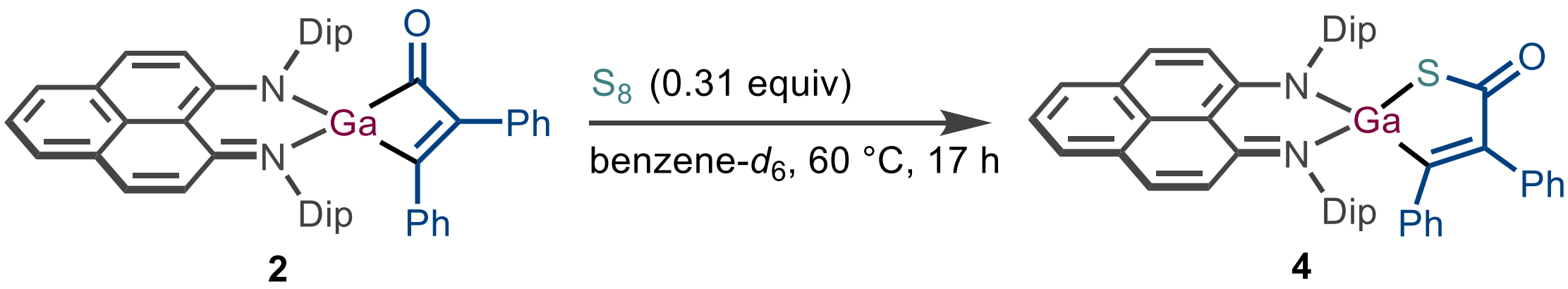

A benzene-d_6 (0.5 mL) solution of **1** (16 mg, 0.020 mmol), S_8 (1.6 mg, 0.0062 mmol) was transferred into a J. Young NMR tube. The tube was then sealed with screwcap, and the mixture was reacted at 60 °C for 17 h under dark. After the reaction, 1,3,5-trimethoxybenzene (TMB) was added to tube under dark as an internal standard. The yield of **2** was determined by the *in situ* comparison of the new resonance at 3.16 ppm (2H) in the ^{1}H NMR spectrum to the TMB (82%).

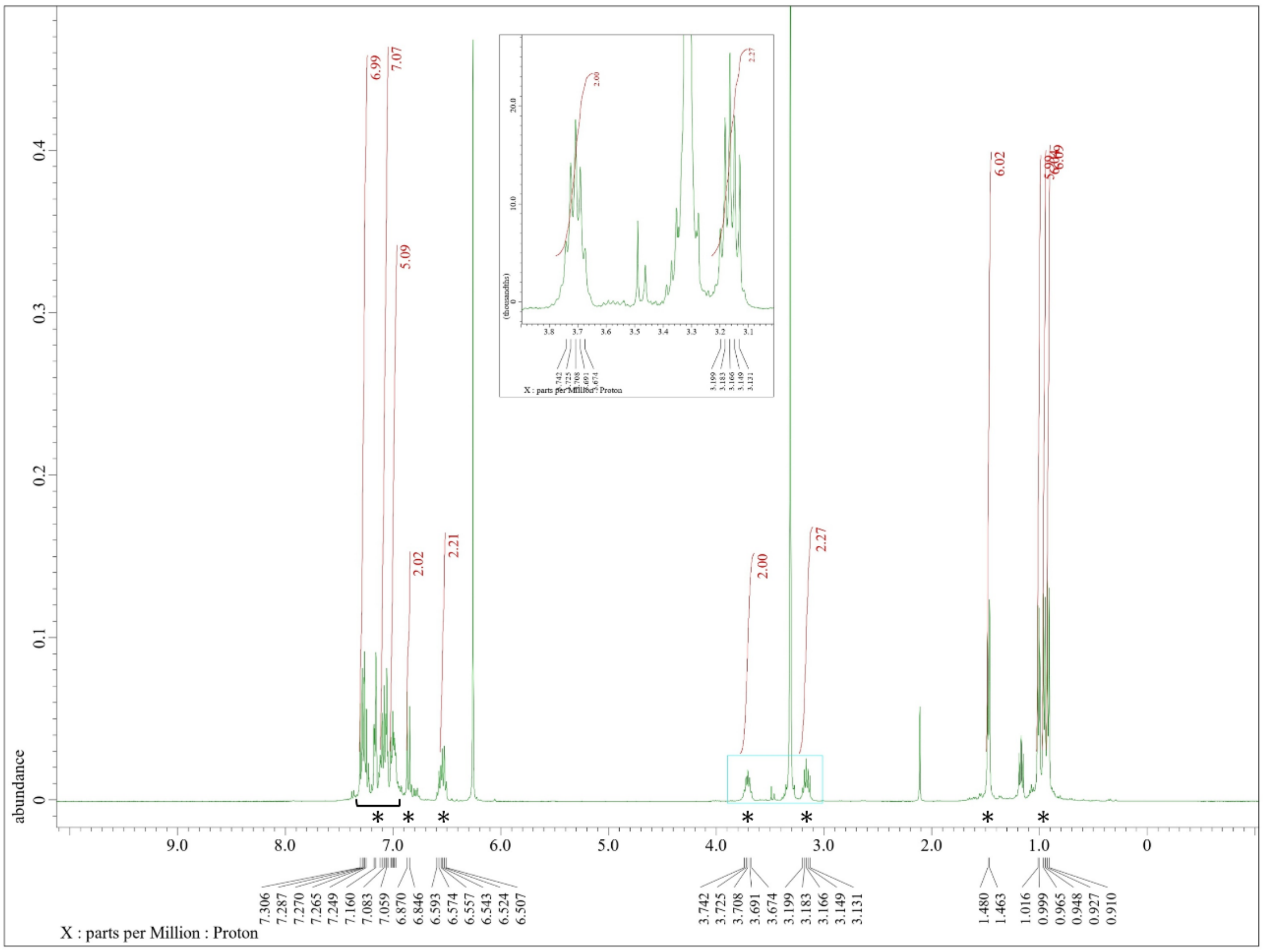

Figure S3. Crude ^{1}H NMR spectra (400 MHz) of the conversion of **2** to **4** in benzene-d_6. Asterisks (*) indicate signals assigned to **4**.

IV. Synthesis of Gallium Complexes

a. Synthesis of 2

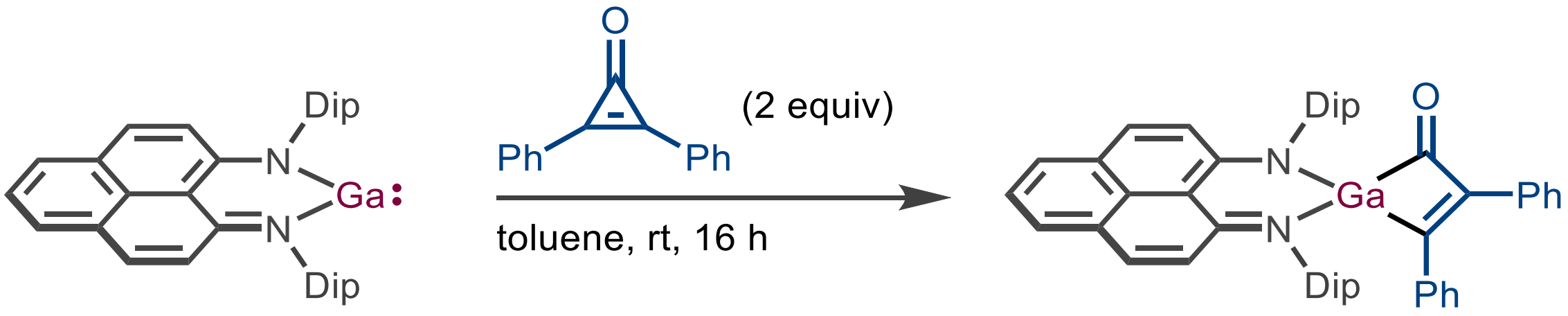

In a nitrogen-filled glovebox, diphenylcyclopropenone (44 mg, 0.21 mmol) was added to a solution of **1** (59 mg, 0.10 mmol) in toluene (1 mL), and the reaction mixture was stirred at rt for 16 h. The solvent was removed under reduced pressure, and the resulting solid was washed with hexane to afford desired product **2** as a red solid (57 mg, 73%). Single crystals suitable for X-ray crystallographic analysis were obtained by slow evaporation of a hexane solution of **2** at rt over 48 h in the glovebox.

Mp 242.0 °C–244.0 °C

^{1}H NMR ($CDCl_3$, 400 MHz) δ: 7.88 (d, J = 7.6 Hz, 2H), 7.72 (d, J = 9.6 Hz, 2H), 7.50 (t, J = 7.6 Hz, 1H), 7.34–7.28 (m, 4H), 7.19 (dd, J = 2.1, 6.9 Hz, 2H), 7.15–7.12 (m, 2H), 7.09 (d, J = 7.3 Hz, 1H), 7.05–7.00 (m, 3H), 6.87–6.83 (m, 4H), 6.62 (dd, J = 2.1, 2.0 Hz, 2H), 3.29 (sep, J = 6.9 Hz, 2H), 3.16 (sep, J = 6.9 Hz, 2H), 1.25 (d, J = 6.6 Hz, 6H), 1.07 (d, J = 6.9 Hz, 6H), 0.91 (d, J = 6.6 Hz, 6H), 0.67 (d, J = 6.6 Hz, 6H)

^{13}C{^{1}H} NMR ($CDCl_3$, 101 MHz) δ: 209.7, 170.4, 162.2, 144.9, 144.1, 141.4, 140.3, 137.2, 134.8, 132.3, 128.9, 128.1, 128.0, 127.9, 127.6, 126.83, 126.79, 126.2, 125.3, 124.7, 122.8, 122.0, 108.2, 28.8, 28.7, 25.6, 25.2, 24.4, 23.5.

IR (KBr, cm^{-1}): 1628 (C=O stretching).

HRMS (DART-TOF) *m/z* $[M+H]^+$ calcd for $C_{52}H_{52}N_2O^{69}Ga$: 789.3330, found: 789.3365.

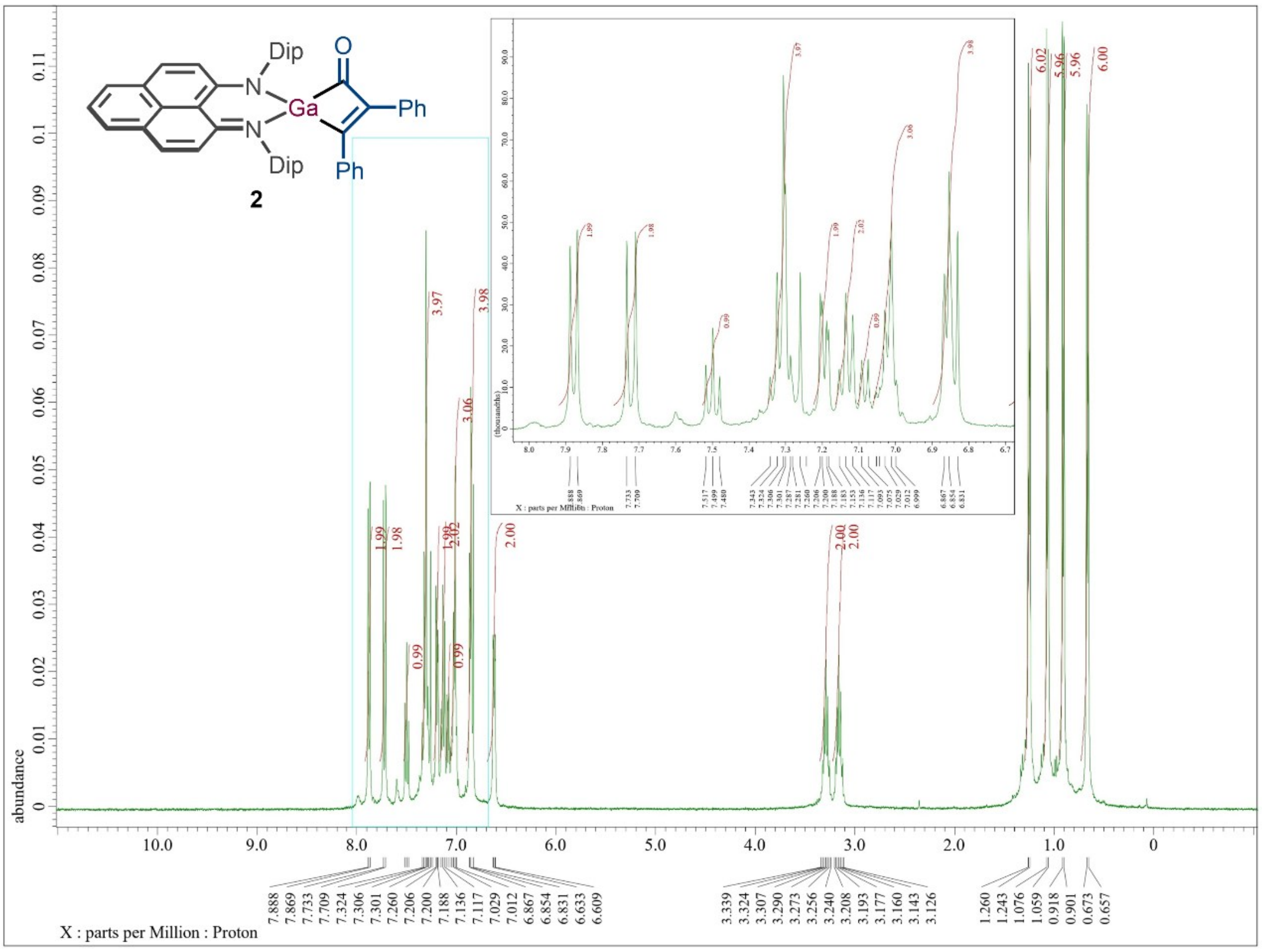

Figure S4. ^{1}H NMR spectra (400 MHz) of **2** in $CDCl_3$.

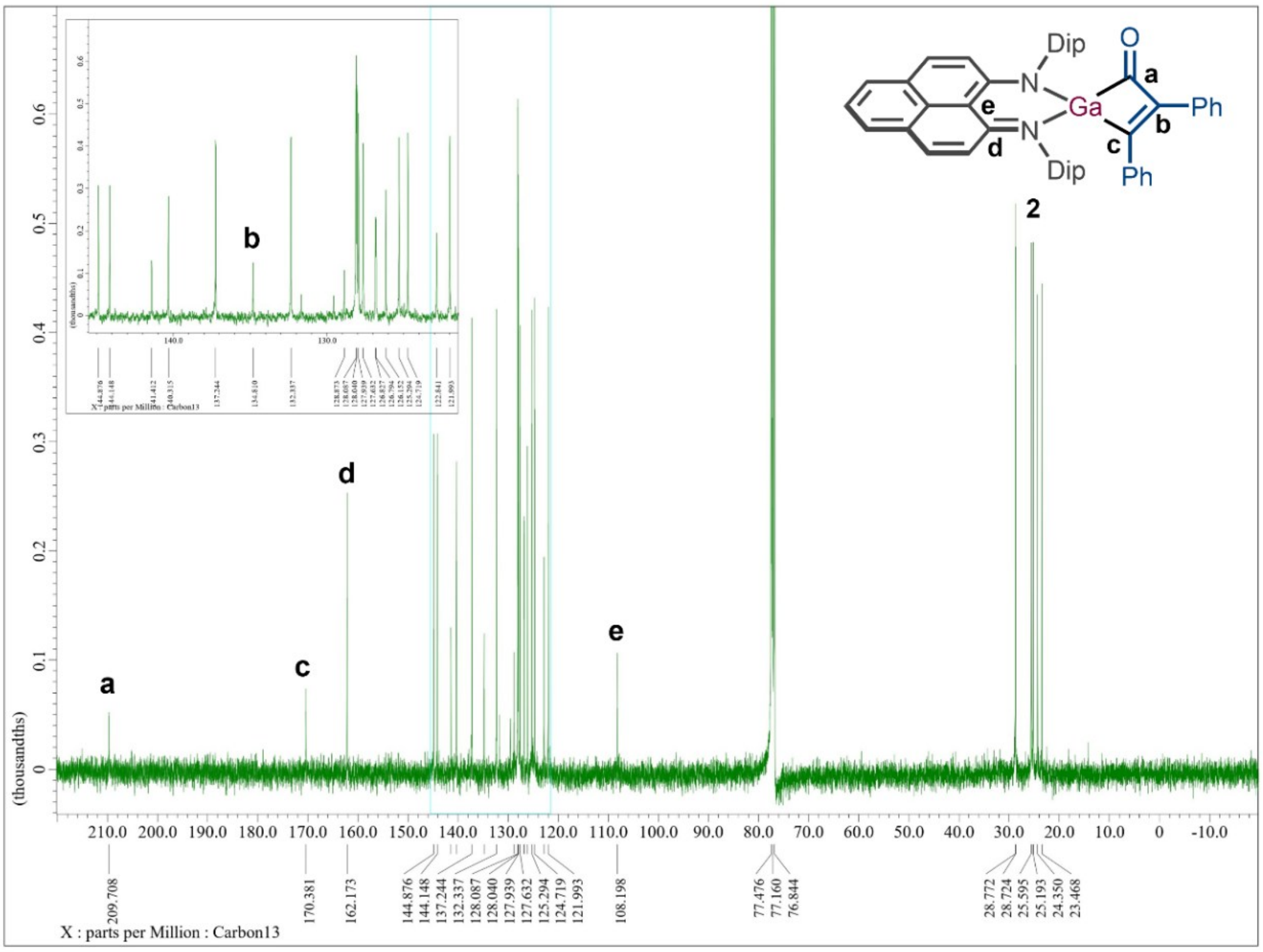

Figure S5. $^{13}C\{^{1}H\}$ NMR spectra (101 MHz) of **2** in $CDCl_3$.

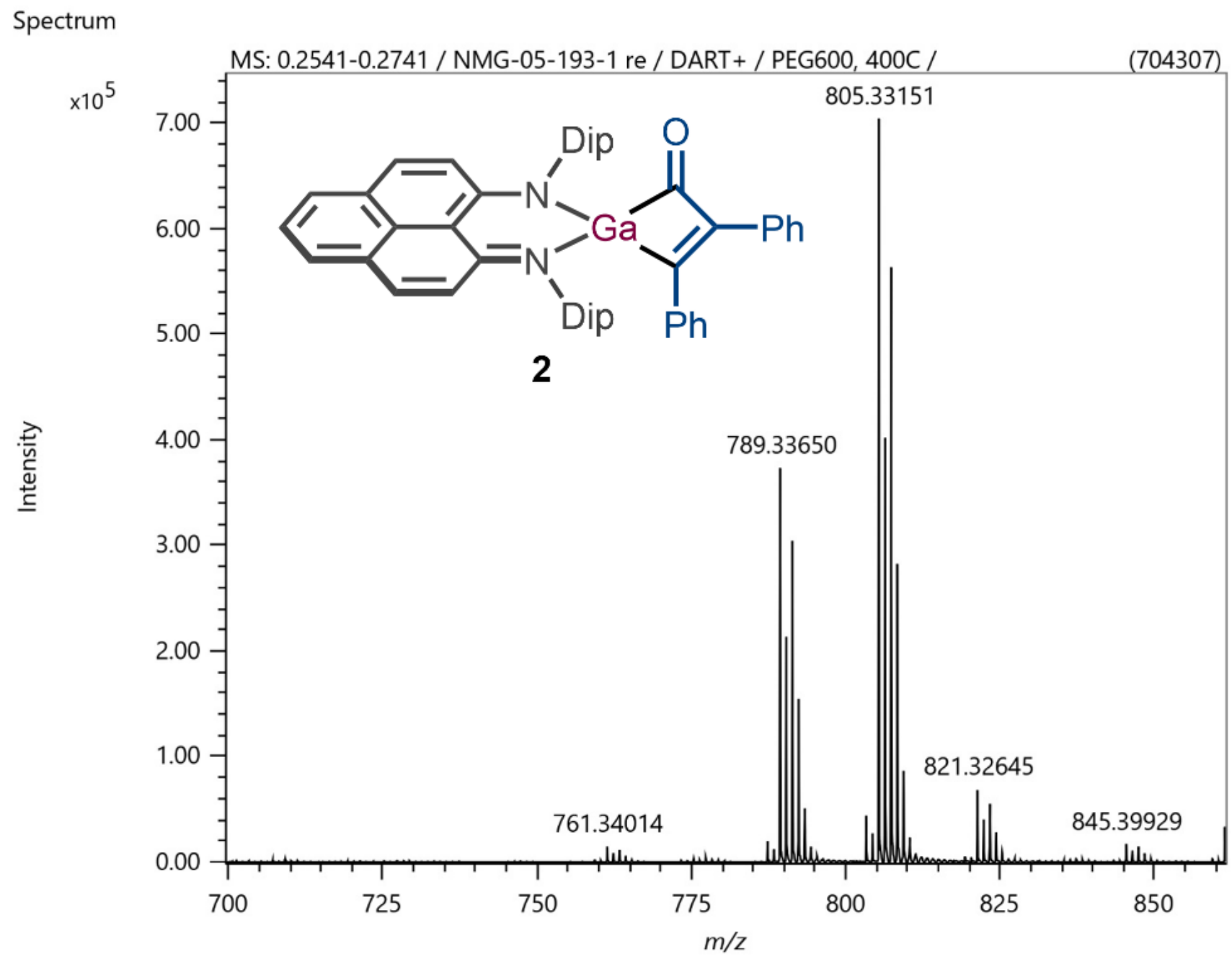

Elemental Composition

Parameters	
Tolerance:	15.00 mDa
Electron:	Odd/Even
Charge:	+1
DBE:	-1.5 - 100.0

Elements Set 1:

Symbol	C	H	Ga	N	O
Min	52	51	1	2	1
Max	52	53	1	2	1

Results

Mass	Intensity	Formula	Calculated Mass	Mass Difference [mDa]	Mass Difference [ppm]	DBE
789.33650	373103.33	C52 H52 N2 O Ga	789.33300	3.51	4.44	28.5

Figure S6. HRMS spectra (DART-TOF) of **2.**

b. Synthesis of 3

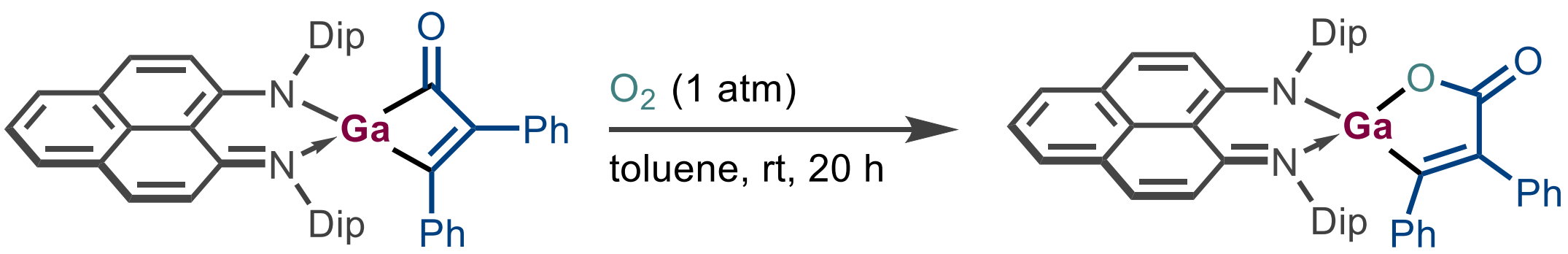

2 **3**

In a nitrogen-filled glovebox, a solution of **2** (159.8 mg, 0.20 mmol) in toluene (3 mL) was transferred to a reaction tube. The solution was degassed by three freeze pomp saw cycles. The tube was then charged with O_2 (1 atm) and the mixture was stirred at rt for 20 h. The solvent was concentrated under reduced pressure, and pentane was added. The mixture was cooled at –35 °C for 1 h, and the resulting solid was collected and washed with pentane (–35 °C) to afford the desired product **3** as an orange solid (83 mg, 51%). Single crystals suitable for crystallographic analysis were obtained by cooling a toluene/pentane solution to –35 °C.

Mp >300 °C.

^{1}H NMR ($CDCl_3$, 400 MHz) δ: 7.98 (d, J = 7.6 Hz, 2H), 7.85 (d, J = 9.4 Hz, 2H), 7.59 (t, J = 7.6 Hz, 1H), 7.41 (d, J = 4.6 Hz, 4H), 7.22-7.19 (m, 2H), 7.02-6.99 (m, 5H), 6.86-6.79 (m, 5H), 5.95 (dd, J = 1.4 Hz, 8.0 Hz, 2H), 3.54 (sep, J = 6.6 Hz, 2H), 2.94 (sep, J = 6.9 Hz, 2H), 1.29 (d, J = 6.9 Hz, 6H), 1.22 (d, J = 6.6 Hz, 6H), 0.76 (d, J = 6.6 Hz, 6H), 0.74 (d, J = 6.9 Hz, 6H).

^{13}C{^{1}H} NMR ($CDCl_3$, 101 MHz) δ: 171.9, 163.4, 149.8, 146.5, 143.2, 141.1, 139.8, 138.0, 136.2, 133.1, 130.4, 128.2, 128.0, 127.4, 127.2, 126.7, 126.2, 125.9, 125.5, 124.6, 123.5, 121.6, 108.4, 29.5, 28.2, 25.5, 25.4, 25.2, 22.6.

IR (KBr, cm^{-1}): 1630 (C=O stretching).

HRMS (DART-TOF) m/z $[M+H]^+$ calcd for $C_{52}H_{52}N_2O_2{}^{69}Ga$: 805.3279, found: 805.3286.

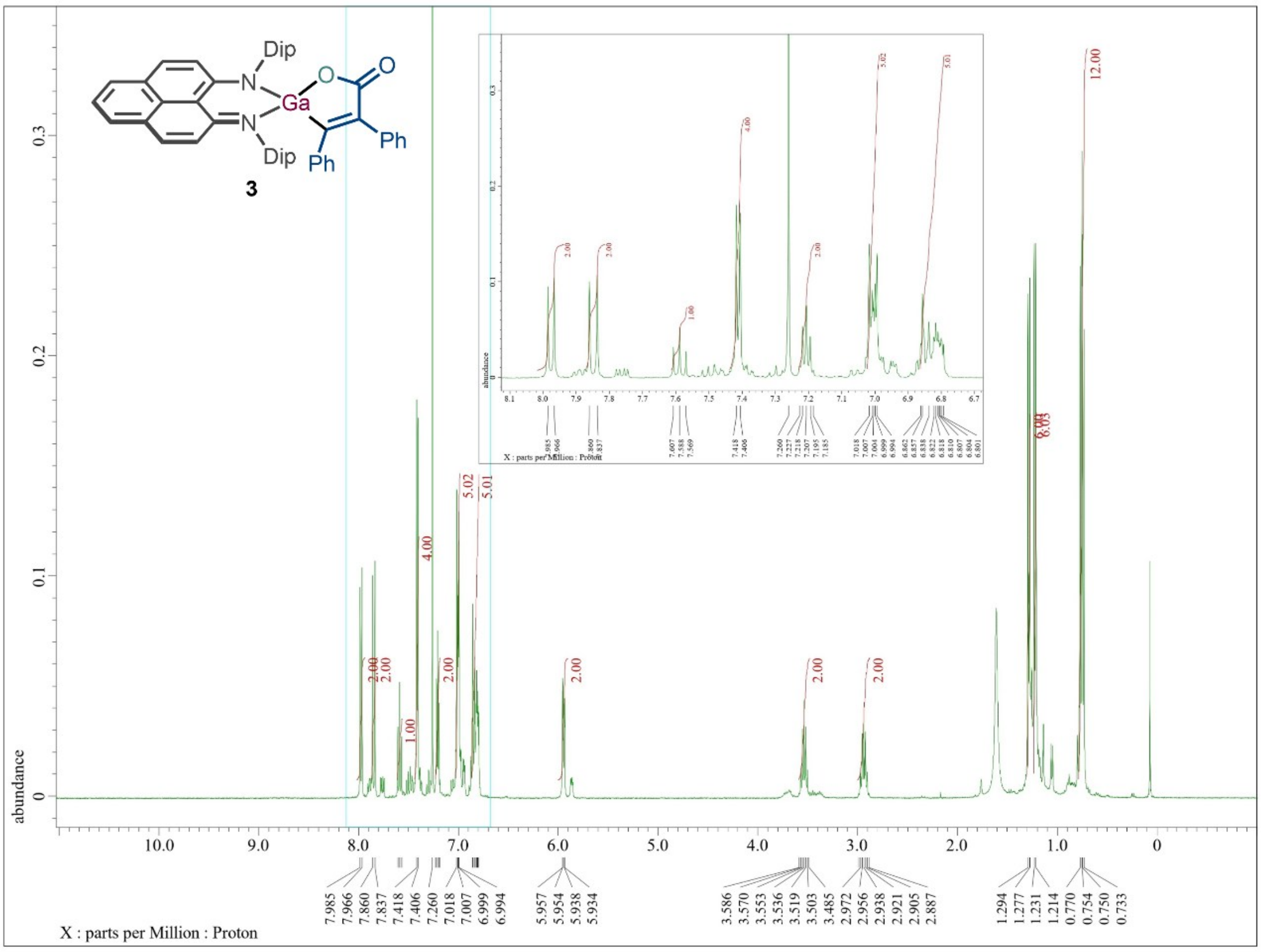

Figure S7. ^{1}H NMR spectra (400 MHz) of **3** in $CDCl_3$.

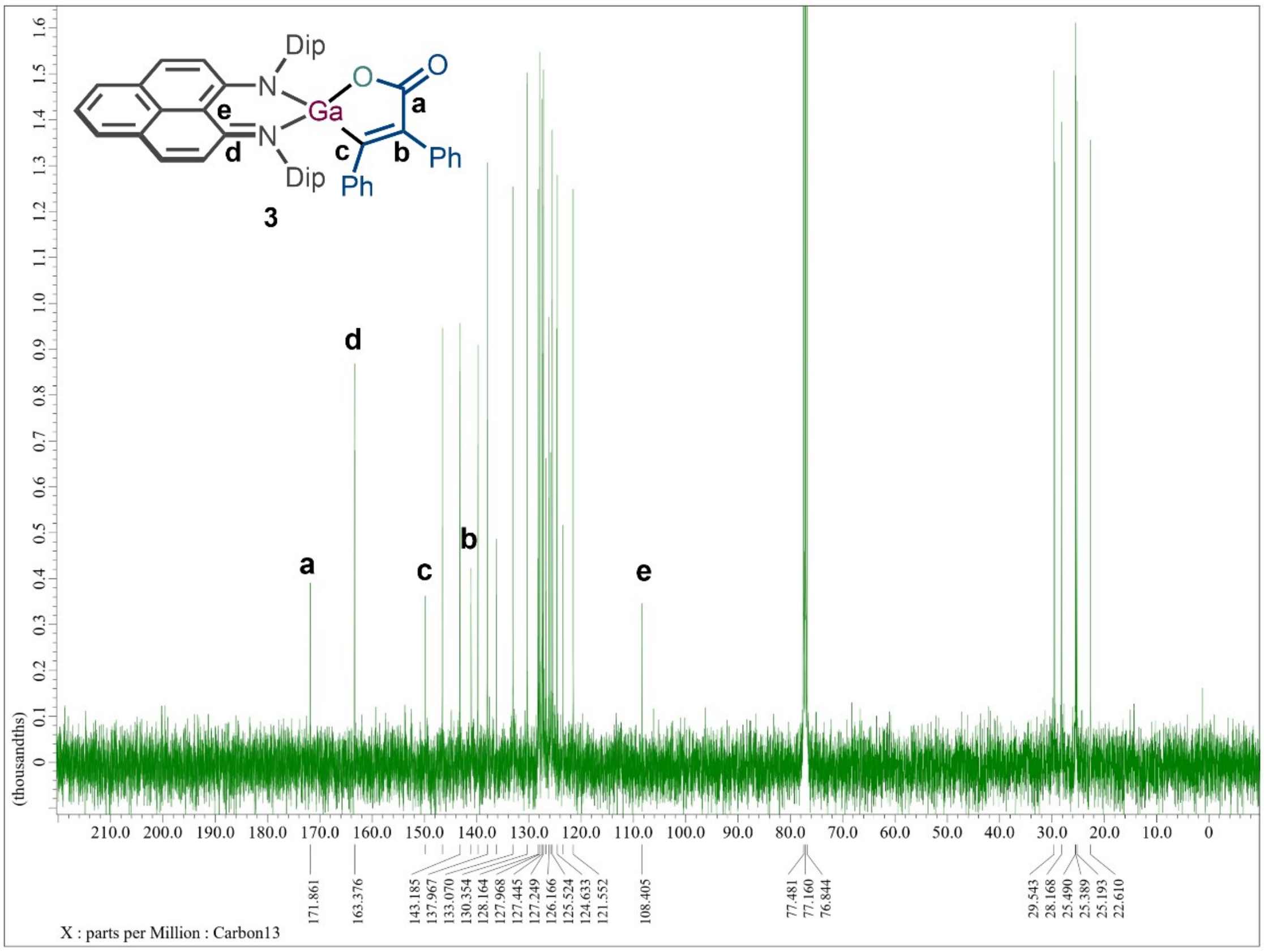

Figure S8. $^{13}C\{^1H\}$ NMR spectra (101 MHz) of **3** in $CDCl_3$.

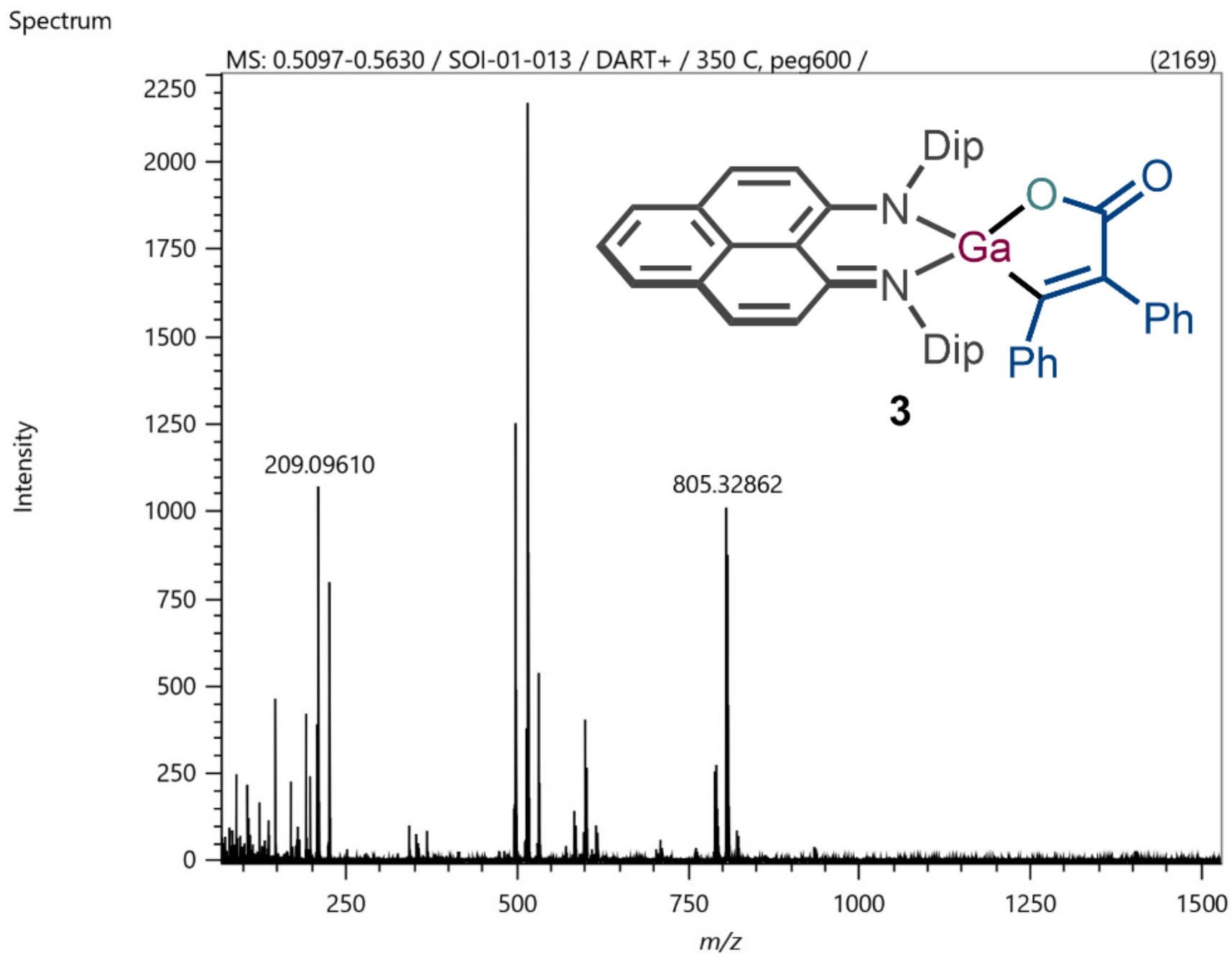

Elemental Composition

Parameters	
Tolerance:	15.00 mDa
Electron:	Odd/Even
Charge:	+1
DBE:	-10.0 - 100.0

Elements Set 1:

Symbol	C	H	O	Ga	Si	N
Min	52	51	1	1	0	2
Max	52	52	2	1	0	2

Results

Mass	Intensity	Formula	Calculated Mass	Mass Difference [mDa]	Mass Difference [ppm]	DBE
805.32862	1010.89	C52 H52 N2 O2 Ga	805.32791	0.71	0.89	28.5

Figure S9. HRMS spectra (DART-TOF) of **3.**

c. Synthesis of 4

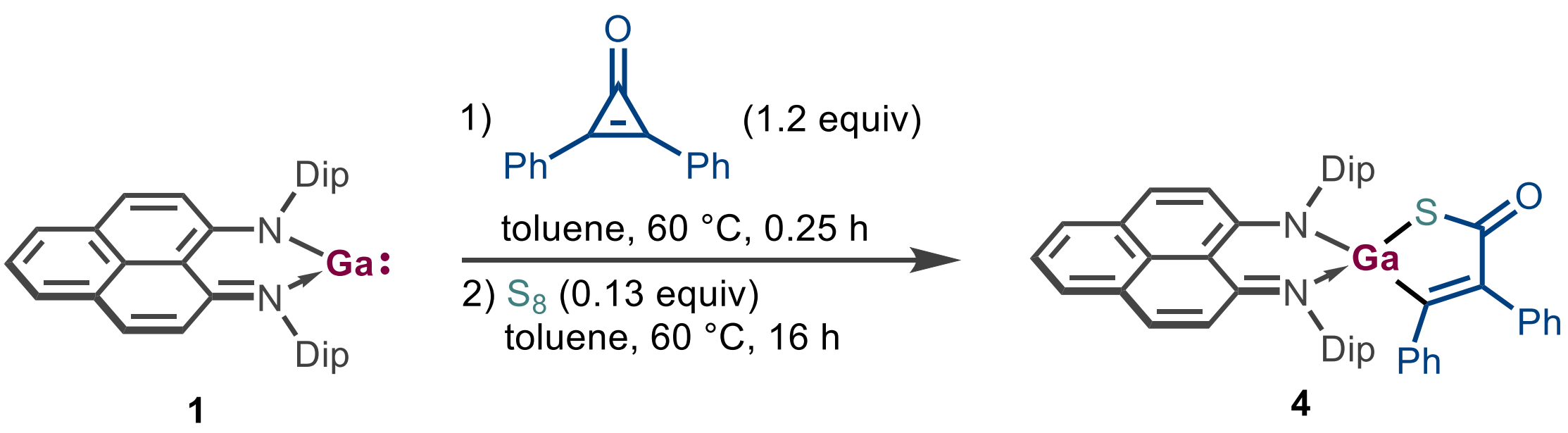

In a nitrogen-filled glovebox, diphenylcyclopropenone (26 mg, 0.12 mmol) was added to a solution of **1** (59 mg, 0.10 mmol) in toluene (1 mL), and the mixture was stirred at 60 °C for 0.25 h. Elemental sulfur (S_8, 33 mg, 0.13 mmol) was then added, and the mixture was stirred at 60 °C in an aluminum heating block for 16 h. The solvent was concentrated under reduced pressure, and pentane was added. The mixture was cooled at –35 °C for 1 h, and the resulting solid was collected and washed with pentane (–35 °C) to afford the desired product **4** as an orange solid (47 mg, 56%). Single crystals

suitable for crystallographic analysis were obtained by cooling a toluene/pentane solution to −35 °C.

Mp 245.0 °C–247.0 °C (decomp.)

^{1}H NMR ($CDCl_3$, 400 MHz) δ: 7.97 (d, J = 7.6 Hz, 2H), 7.79 (d, J = 9.6 Hz, 2H), 7.59 (t, J = 7.8 Hz,1H), 7.42–7.35 (m, 4H), 7.30–7.24 (m, 5H), 6.98–6.89 (m, 5H), 6.83–6.77 (m, 4H), 3.42 (sep, J = 6.4 Hz, 2H), 2.93 (sep, J = 6.4 Hz, 2H), 1.36 (d, J = 6.9 Hz, 6H), 0.96 (d, J = 6.6 Hz, 6H), 0.86 (d, J = 6.6 Hz, 6H), 0.82 (d, J = 6.9 Hz, 6H).

$^{13}C\{^{1}H\}$ NMR ($CDCl_3$, 101 MHz) δ: 202.3, 163.4, 145.1, 144.6, 140.1, 139.2, 139.1, 137.5, 132.7, 131.3, 129.2, 128.7, 128.5, 128.0, 127.9, 127.8, 127.1, 126.3, 125.5, 124.8, 123.2, 122.5, 108.1, 29.5, 28.4, 25.4, 24.8, 24.3, 24.3.

IR (KBr, cm^{-1}): 1627 (C=O stretching).

HRMS (DART-TOF) m/z $[M+H]^+$ calcd for $C_{52}H_{52}N_2OS^{69}Ga$: 821.3051, found: 821.3057.

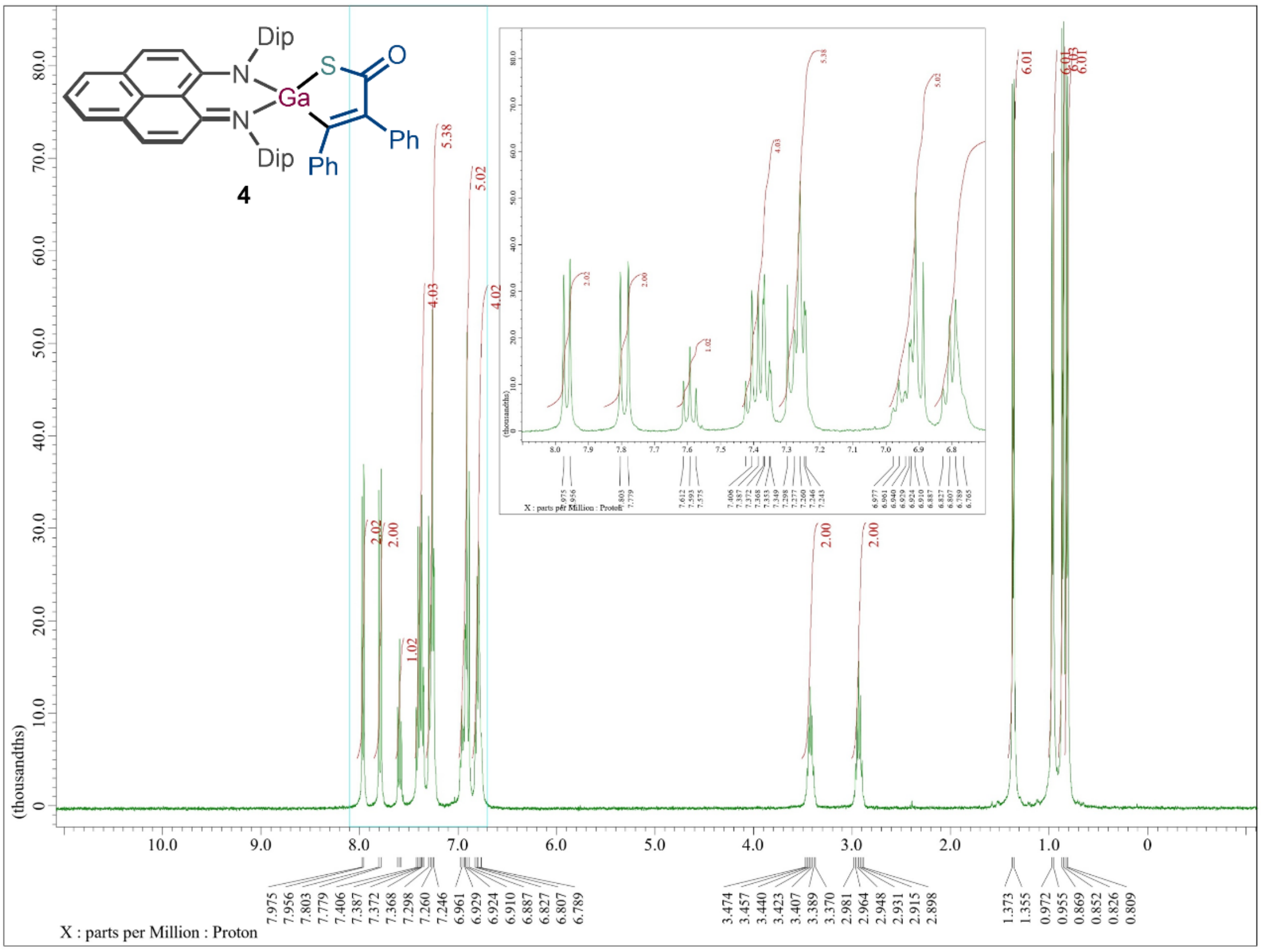

Figure S10. ^{1}H NMR spectra (400 MHz) of **4** in $CDCl_3$.

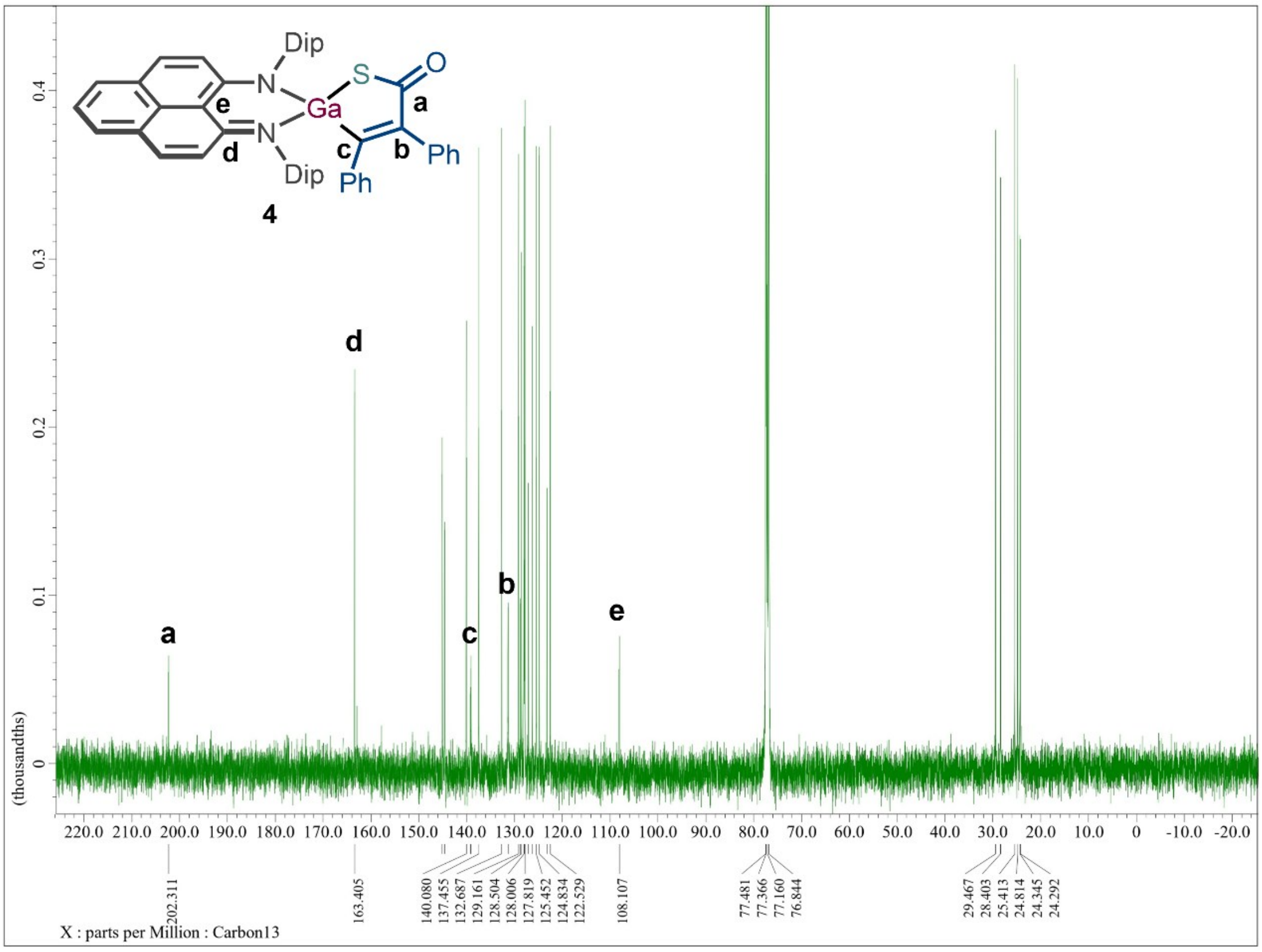

Figure S11. $^{13}C\{^1H\}$ NMR spectra (101 MHz) of **4** in $CDCl_3$.

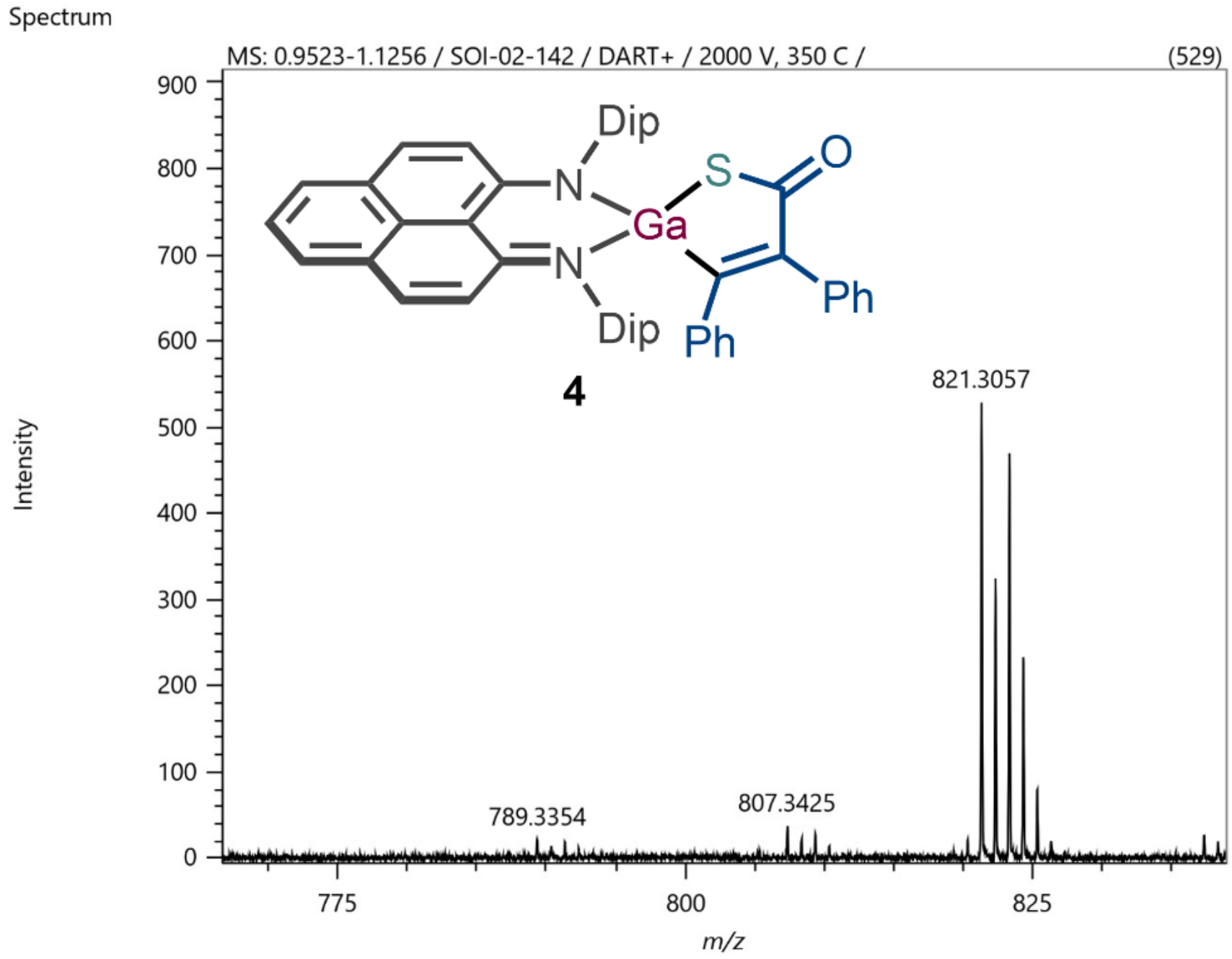

Elemental Composition

Parameters	
Tolerance:	100.00 ppm
Electron:	Odd/Even
Charge:	+1
DBE:	-99.0 - 999.0

Elements Set 1:

Symbol	C	H	N	Ga	O	S
Min	52	51	2	1	1	1
Max	52	52	2	1	1	1

Results

Mass	Intensity	Formula	Calculated Mass	Mass Difference [mDa]	Mass Difference [ppm]	DBE
821.30570	528.59	C52 H52 N2 O S Ga	821.30507	0.63	0.77	28.5

Figure S12. HRMS spectra (DART-TOF) of **4.**

d. Larger-Scale Synthesis of 2

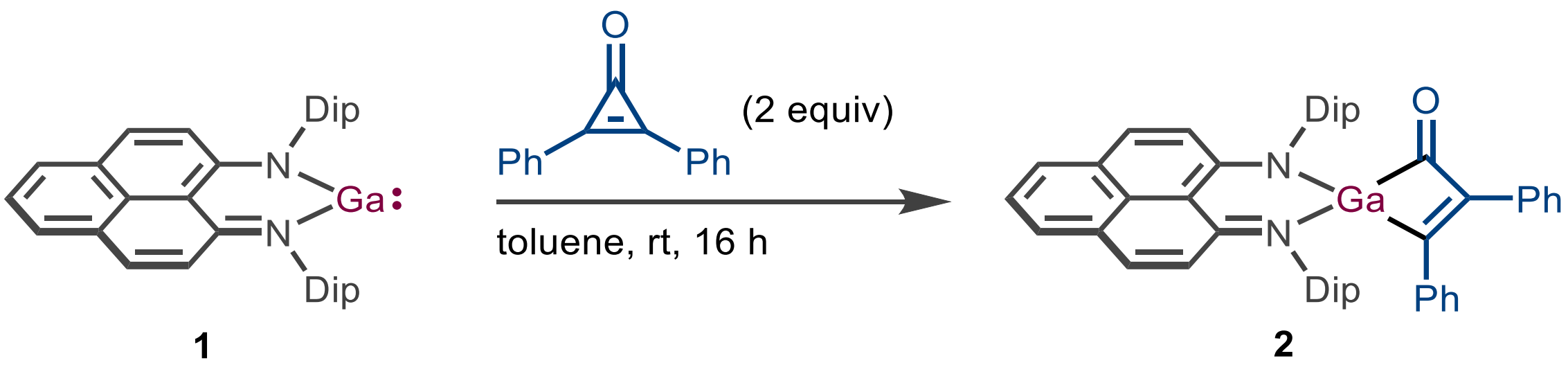

In a nitrogen-filled glovebox, diphenylcyclopropenone (0.71 g, 3.4 mmol) was added to a toluene (20 mL) solution of **1** (1.0 g, 1.7 mmol), and the mixture was stirred at rt for 16 h. The solvent was removed under reduced pressure. The resulting solid was precipitated with hexane at –60 °C. The precipitated was collected, washed with MeCN, and dried under reduced pressure to afford desired product as a red solid **2** (0.47 g, 35%).

V. Trapping Experiment of Ketene Intermediate INT2

General Procedure

In a glovebox filled with nitrogen, diphenylcyclopropenone (0.02 mmol) and trapping reagent (0.03 mmol) were added to a toluene-d_8 (0.5 mL) solution of **1** (0.02 mmol) at −78 °C. After stirring for 1 h, the mixture was warmed to room temperature over 5 h. Then, 1,3,5-trimethoxybenzene (TMB) was added to tube under dark as an internal standard. Oxidative addition product **2** was obtained in all cases, and no ketene-derived products were detected.

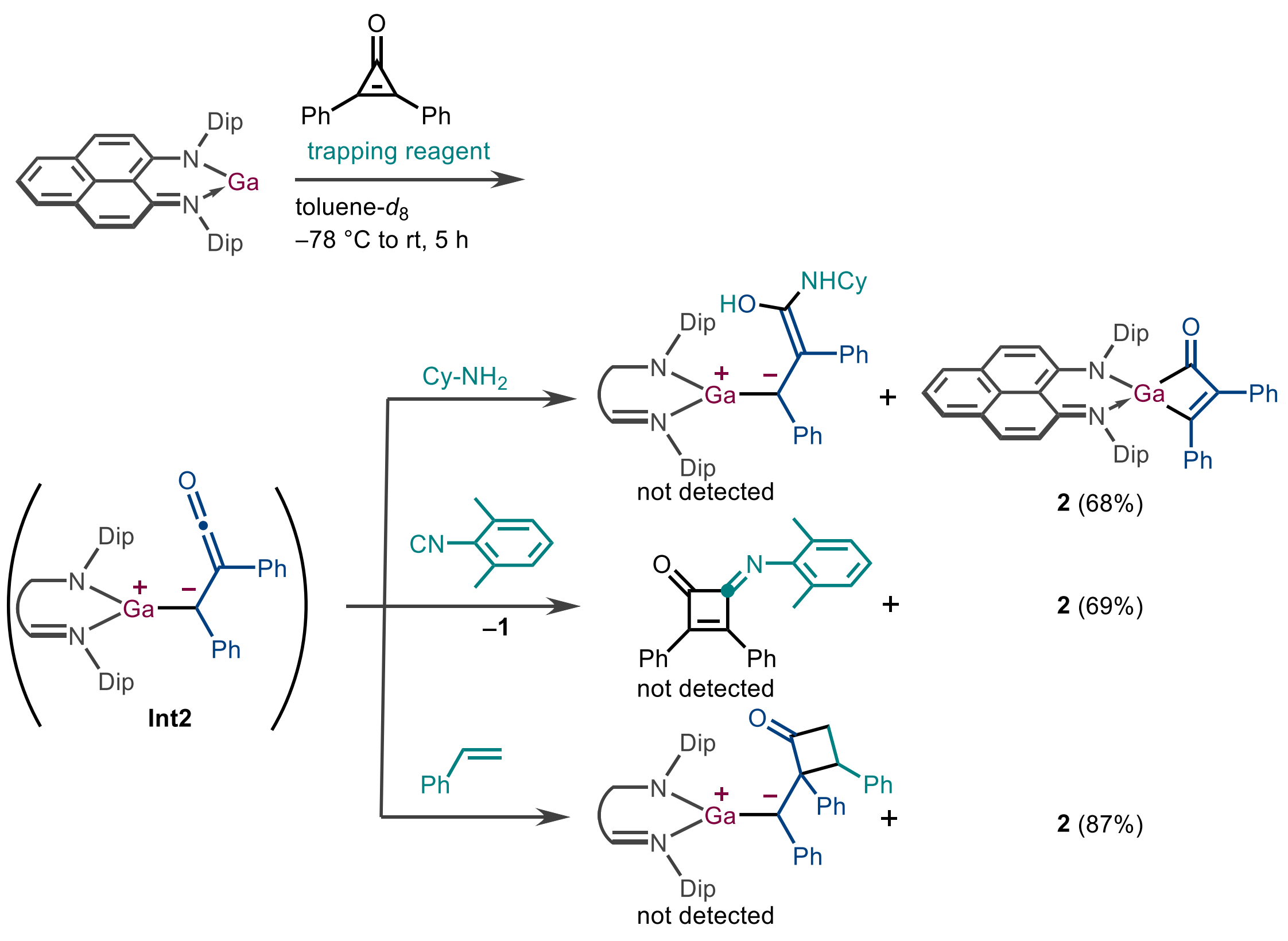

VI. Crystallographic Information

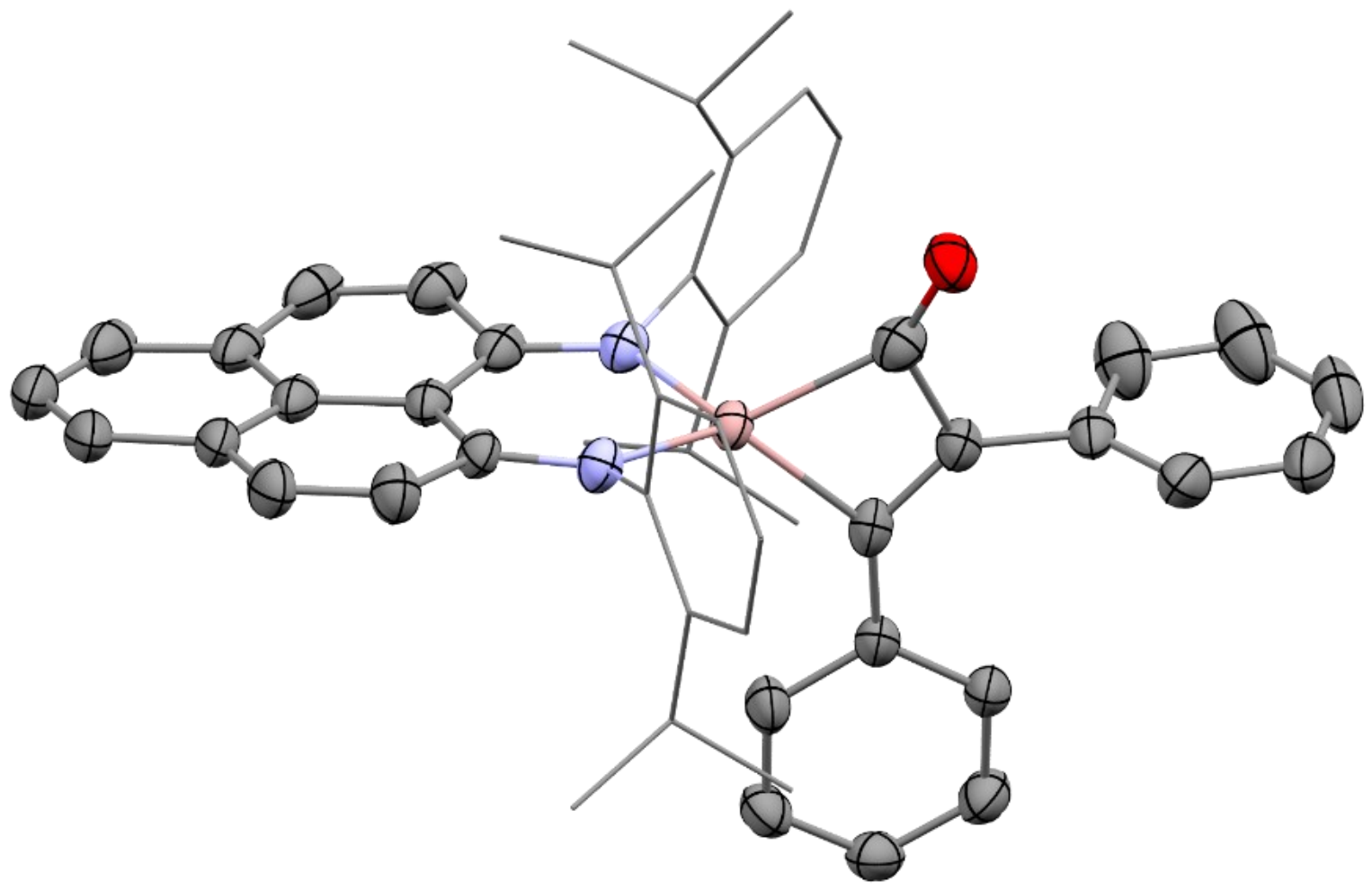

Figure S13. ORTEP drawing of **2**.[a]
[a]Crystal data for **2**, orthorhombic, space group *P n a* 2_1 (no. 33), *a* = 19.0175(5) Å, *b* = 11.9149(4) Å, *c* = 18.4607(6) Å, *α* = *β* = *γ* = 90°, *V* = 4183.0(2) Å^3, *T* = 123 K, *Z* = 4, *R*1 (*wR*2) = 0.0382 (0.0987) for 513 parameters and 6997 unique reflections. GOF = 1.0231. CCDC 2553834.

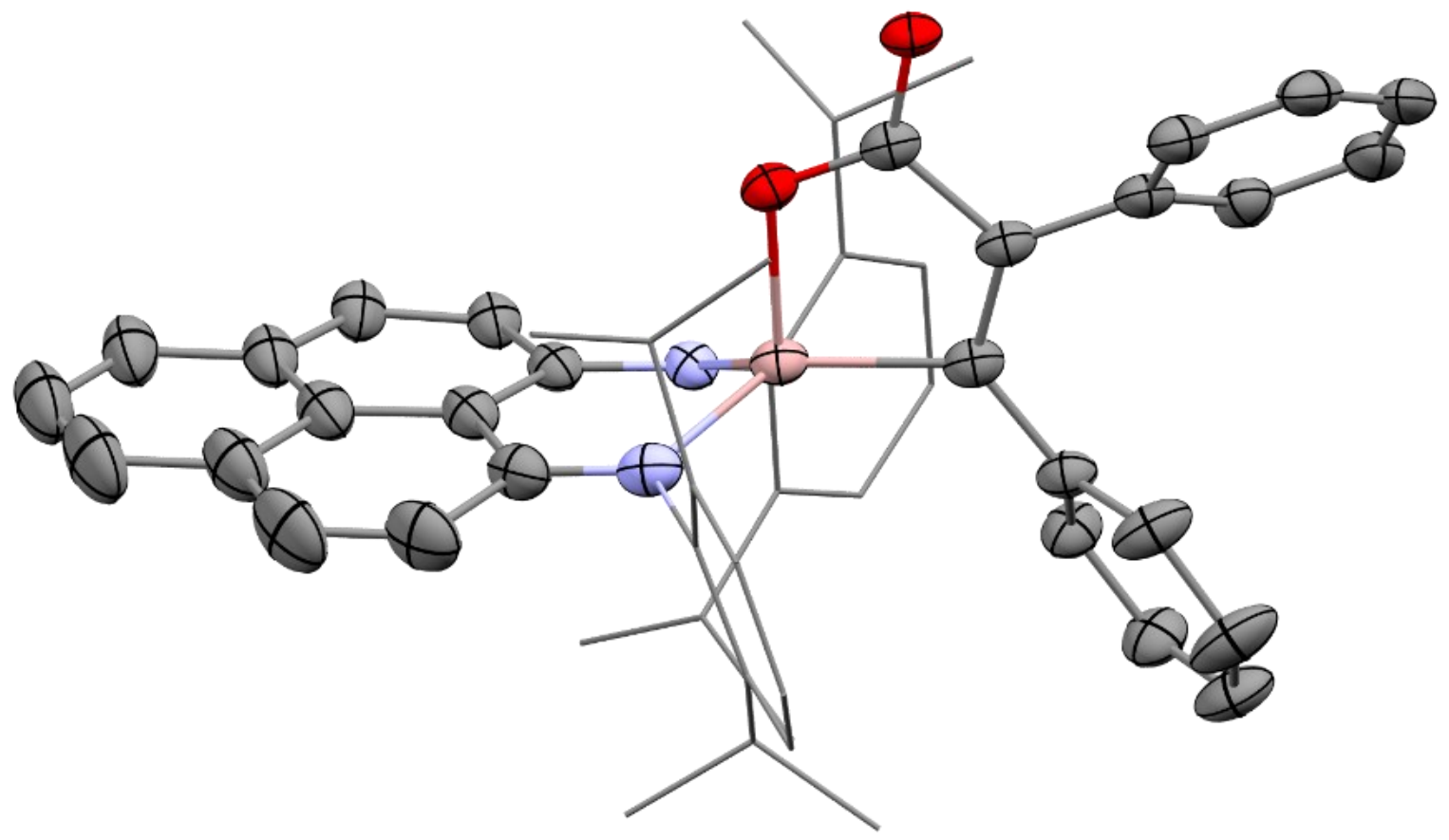

Figure S14. ORTEP drawing of **3**.[a]
[a]Crystal data for **3**, monoclinic, space group *P* 2_1/*c* (no. 14), *a* = 19.2782(3) Å, *b* = 37.3948(5) Å, *c* = 15.55024(18) Å, *α* = 90°, *β* = 102.3371(12)°, *γ* = 90°, *V* = 10951.4(2) Å^3, *T* = 123.00 K, *Z* = 4, *R*1 (*wR*2) = 0.0482 (0.1333) for 1098 parameters and 21728 unique reflections. GOF = 1.062. CCDC 2553835.

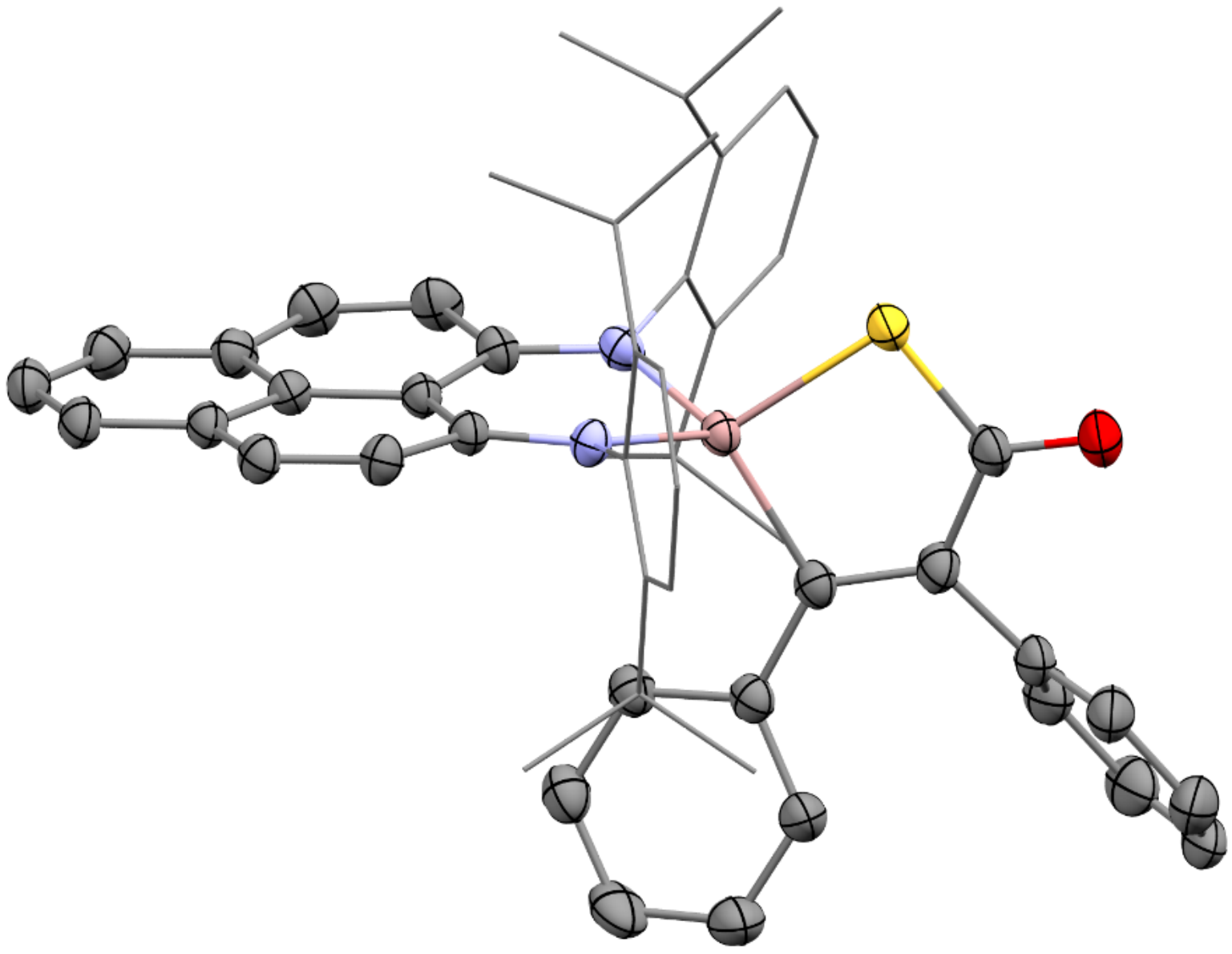

Figure S15. ORTEP drawing of **4**.[a]
[a]Crystal data for **4**, triclinic, space group $P\bar{1}$ (no. 2), a = 15.2298(2) Å, b = 17.8150(2) Å, c = 19.8750(2) Å, α = 86.5020(10)°, β = 85.4090(10)°, γ = 86.4870(10)°, V = 5356.52(11) Å^3, T = 123.15 K, Z = 2, R1 (wR2) = 0.0761 (0.2339) for 1253 parameters and 21646 unique reflections. GOF = 1.045. CCDC 2553836.

VII. References

(1) Frisch, M. J.; Trucks, G. W.; Schlegel, H. B.; Scuseria, G. E.; Robb, M. A.; Cheeseman, J. R.; Scalmani, G.; Barone, V.; Mennucci, B.; Petersson, G. A.; Nakatsuji, H.; Caricato, M.; Li, X.; Hratchian, H. P.; Izmaylov, A. F.; Bloino, J.; Zheng, G.; Sonnenberg, J. L.; Hada, M.; Ehara, M.; Toyota, K.; Fukuda, R.; Hasegawa, J.; Ishida, M.; Nakajima, T.; Honda, Y.; Kitao, O.; Nakai, H.; Vreven, T.; Montgomery, J. A., Jr.; Peralta, J. E.; Ogliaro, F.; Bearpark, M.; Heyd, J. J.; Brothers, E.; Kudin, K. N.; Staroverov, V. N.; Keith, T.; Kobayashi, R.; Normand, J.; Raghavachari, K.; Rendell, A.; Burant, J. C.; Iyengar, S. S.; Tomasi, J.; Cossi, M.; Rega, N.; Millam, N. J.; Klene, M.; Knox, J. E.; Cross, J. B.; Bakken, V.; Adamo, C.; Jaramillo, J.; Gomperts, R.; Stratmann, R. E.; Yazyev, O.; Austin, A. J.; Cammi, R.; Pomelli, C.; Ochterski, J. W.; Martin, R. L.; Morokuma, K.; Zakrzewski, V. G.; Voth, G. A.; Salvador, P.; Dannenberg, J. J.; Dapprich, S.; Daniels, A. D.; Farkas, Ö .; Foresman, J. B.; Ortiz, J. V.; Cioslowski, J.; Fox, D. J. Gaussian 09, Revision D.01; Gaussian, Inc.: Wallingford, CT, 2013.
(2) (a) Reed, A. E.; Weinhold, F. Natural localized molecular orbitals. *J. Chem. Phys.* **1985**, *83*, 1736–1740; (b) Reed, A. E.; Weinhold, F. Natural Bond Orbital Analysis of Internal Rotation Barriers and Related Phenomena. *Isr. J. Chem*. **1991**, *31*, 277–285; (c) Reed, A. E.; Curtiss, L. A.; Weinhold, F. Intermolecular interactions from a natural bond orbital, donor-acceptor viewpoint. *Chem. Rev.* **1988**, *88*, 899–926.
(3) Glendening, E. D.; Reed, A. E.; Carpenter, J. E.; Weinhold, F. NBO Version 3.1.
(4) Kodama, T.; Mukai, N.; Tobisu, M. Synthesis, Structure, and Reactivity of a Gallylene Derivative Bearing a Phenalenyl-Based Ligand. *Inorg. Chem.* **2023**, *62*, 6554−6559. DOI: 10.1021/acs.inorgchem.3c00697.
(5) Dey, S.; Ghosh, P. Accessing Heteroannular Benzoxazole and Benzimidazole Scaffolds via Carbodiimides Using Azide–Isocyanide Cross-Coupling as Catalyzed by Mesoionic Singlet Palladium Carbene Complexes Derived from a Phenothiazine Moiety. *ACS Omega* **2023**, *8*, 11039−11064. DOI: 10.1021/acsomega.2c07875.

VIII. Cartesian Coordinates of Optimized Structure

1

7	-1.381057000	-0.273044000	0.013790000
7	1.381057000	-0.273045000	0.013790000
6	0.000000000	1.735053000	-0.003425000
6	1.265635000	1.051832000	-0.000118000
6	1.208803000	3.907445000	-0.022755000
6	0.000000000	3.164007000	-0.015619000
6	-1.265635000	1.051833000	-0.000117000
6	-1.208803000	3.907445000	-0.022752000
6	-2.682023000	-0.852333000	0.004105000
6	-3.305780000	-1.164000000	1.220513000
6	2.682023000	-0.852334000	0.004106000
6	-2.436709000	3.197180000	-0.021617000
1	-3.364256000	3.759646000	-0.028589000
6	3.305779000	-1.164000000	1.220515000
6	2.463485000	1.850031000	-0.012727000
1	3.410036000	1.328978000	-0.012764000
6	2.436710000	3.197180000	-0.021623000
1	3.364256000	3.759645000	-0.028597000
6	1.190579000	5.302012000	-0.032788000
1	2.137798000	5.829871000	-0.037682000
6	-1.190579000	5.302012000	-0.032786000
1	-2.137797000	5.829871000	-0.037677000
6	-2.463484000	1.850031000	-0.012722000
1	-3.410036000	1.328978000	-0.012758000
6	3.274807000	-1.187052000	-1.221732000
6	4.534849000	-1.812649000	1.186168000
1	5.029168000	-2.062216000	2.118053000
6	5.134640000	-2.147098000	-0.014897000
6	0.000000000	6.005358000	-0.037142000
1	0.000000000	7.087689000	-0.044824000
6	-3.274806000	-1.187052000	-1.221734000
6	-4.534850000	-1.812649000	1.186165000
1	-5.029169000	-2.062216000	2.118049000
6	4.504549000	-1.835120000	-1.206469000
1	4.975037000	-2.102448000	-2.145737000
6	-5.134640000	-2.147098000	-0.014900000
6	-4.504548000	-1.835120000	-1.206471000
1	-4.975035000	-2.102448000	-2.145740000
1	6.091853000	-2.654363000	-0.022244000
1	-6.091853000	-2.654362000	-0.022248000
6	2.605274000	-0.868105000	-2.539344000
1	1.689869000	-0.316219000	-2.319652000
6	2.206120000	-2.144363000	-3.274865000
6	3.478833000	0.025507000	-3.414096000
1	3.737059000	0.951644000	-2.897518000
1	4.409358000	-0.475090000	-3.691555000
1	2.954594000	0.285508000	-4.336387000
1	3.084264000	-2.741661000	-3.531617000
1	1.679460000	-1.905145000	-4.201714000
1	1.551316000	-2.764215000	-2.658766000
6	2.670726000	-0.818707000	2.548438000
1	1.749655000	-0.271257000	2.341553000
6	3.566644000	0.092294000	3.381752000
6	2.291864000	-2.079776000	3.320020000
1	1.620292000	-2.711332000	2.734655000
1	3.176766000	-2.672255000	3.564460000
1	1.791133000	-1.821646000	4.256114000
1	4.504138000	-0.402994000	3.644896000
1	3.066613000	0.371163000	4.311930000
1	3.811194000	1.007663000	2.839970000
6	-2.605272000	-0.868105000	-2.539345000
1	-1.689864000	-0.316223000	-2.319652000
6	-3.478828000	0.025513000	-3.414094000
6	-2.206124000	-2.144363000	-3.274869000
1	-1.551322000	-2.764219000	-2.658773000
1	-3.084270000	-2.741656000	-3.531623000
1	-1.679463000	-1.905144000	-4.201718000
1	-4.409356000	-0.475080000	-3.691553000
1	-2.954589000	0.285513000	-4.336385000
1	-3.737049000	0.951650000	-2.897514000
6	-2.670728000	-0.818708000	2.548437000
1	-1.749657000	-0.271257000	2.341552000
6	-2.291865000	-2.079777000	3.320018000
6	-3.566646000	0.092292000	3.381751000
1	-3.811197000	1.007662000	2.839970000
1	-4.504141000	-0.402997000	3.644894000
1	-3.066616000	0.371160000	4.311930000
1	-3.176767000	-2.672257000	3.564457000
1	-1.791135000	-1.821648000	4.256113000
1	-1.620292000	-2.711332000	2.734653000
31	0.000000000	-1.811743000	0.049648000

Method: PBE0/def2-SVP opt freq Empirical Dispersion=GD3BJ
imaginary frequencies: 0
Sum of electronic and zero-point Energies= –3467.031971 A.U.

Diphenylcyclopropenone

6	-0.683040000	0.833689000	-0.000531000
6	0.683346000	0.834088000	0.000141000
6	-0.000202000	2.087867000	-0.000381000
8	0.000134000	3.294205000	-0.000289000
6	1.925685000	0.100140000	-0.003701000
6	3.127439000	0.827158000	0.044104000
6	1.974041000	-1.302476000	-0.056815000
6	4.349271000	0.163551000	0.042530000
1	3.080549000	1.917830000	0.082201000
6	3.197559000	-1.961488000	-0.059888000
1	1.044857000	-1.872941000	-0.101015000
6	4.385946000	-1.230275000	-0.009035000
1	5.279526000	0.734705000	0.080891000
1	3.228647000	-3.052548000	-0.102701000
1	5.346247000	-1.751459000	-0.010749000
6	-1.925536000	0.099897000	0.003334000
6	-3.127098000	0.827221000	-0.043269000
6	-1.974281000	-1.302743000	0.056224000

6	-4.349028000	0.163903000	-0.041732000	6	-4.386188000	-1.229970000	0.009418000
1	-3.080094000	1.917904000	-0.080932000	1	-5.279039000	0.735502000	-0.079469000
6	-3.198021000	-1.961496000	0.059673000	1	-3.229114000	-3.052561000	0.102225000
1	-1.045333000	-1.873701000	0.100018000	1	-5.346687000	-1.750765000	0.011409000

Method: PBE0/def2-SVP opt freq Empirical Dispersion=GD3BJ
imaginary frequencies: 0
Sum of electronic and zero-point Energies= –651.421702 A.U.

Int1

31	-0.146680000	0.453248000	0.368346000	6	4.052319000	-2.128970000	-0.381412000
6	-3.950414000	0.751615000	0.900606000	6	3.197564000	3.115175000	-3.091929000
8	-2.616356000	1.138923000	3.039685000	1	2.872412000	4.137907000	-3.340171000
7	1.888985000	0.831715000	0.237772000	1	4.212807000	3.174815000	-2.671324000
6	2.842605000	-1.380974000	-0.187819000	1	3.253810000	2.556908000	-4.038987000
7	0.402693000	-1.492973000	-0.077679000	6	2.203268000	5.002740000	0.689244000
6	-1.315629000	-2.886689000	0.968185000	1	2.263349000	6.088015000	0.803003000
6	1.604638000	-3.474572000	-0.665467000	6	-3.224638000	-3.614746000	-0.346755000
1	0.645444000	-3.979923000	-0.776975000	1	-4.173070000	-4.151248000	-0.412883000
6	2.946727000	0.032850000	0.094466000	6	-0.587879000	-2.841271000	2.297813000
6	-4.847512000	-0.264166000	0.405921000	1	0.276654000	-2.171944000	2.171923000
6	-2.968943000	4.139266000	-2.320001000	6	-0.045920000	-4.219989000	2.677616000
1	-3.436849000	4.277215000	-3.297435000	1	-0.864780000	-4.943051000	2.823893000
6	5.335726000	-1.516667000	-0.263839000	1	0.619867000	-4.618388000	1.897346000
6	2.218415000	3.043976000	-0.746043000	1	0.525523000	-4.167637000	3.617649000
6	-2.527527000	-3.572892000	0.855444000	6	-5.241511000	-0.323629000	-0.940008000
1	-2.937961000	-4.077566000	1.733797000	1	-4.848230000	0.407586000	-1.647805000
6	4.268599000	0.606257000	0.209296000	6	-5.331109000	-1.227101000	1.306385000
1	4.334378000	1.669749000	0.439024000	1	-5.004356000	-1.184477000	2.347608000
6	-2.809269000	2.937828000	-0.222398000	6	-2.723644000	-2.946480000	-1.457390000
6	2.067504000	2.232789000	0.401656000	1	-3.289176000	-2.958916000	-2.392632000
6	-1.934644000	4.983750000	-1.913616000	6	3.158803000	1.972441000	3.730126000
1	-1.590987000	5.781284000	-2.577056000	1	4.014665000	1.658527000	3.113535000
6	1.579500000	-2.075752000	-0.300880000	1	3.369028000	2.986832000	4.106157000
6	-1.461191000	-2.253475000	3.406273000	1	3.100010000	1.298344000	4.598949000
1	-1.808981000	-1.236688000	3.167017000	6	-1.336391000	4.810285000	-0.664513000
1	-2.347682000	-2.880301000	3.596145000	1	-0.516960000	5.458267000	-0.348707000
1	-0.893729000	-2.198460000	4.348713000	6	-1.769839000	3.794469000	0.178702000
6	6.457963000	-3.618432000	-0.758354000	1	-1.295719000	3.638442000	1.149762000
1	7.377956000	-4.188665000	-0.900397000	6	4.027078000	-3.518213000	-0.706031000
6	1.971315000	2.800132000	1.689206000	6	-1.513055000	-2.250787000	-1.396612000
6	1.853220000	1.938759000	2.932334000	6	2.235810000	2.424501000	-2.130756000
1	1.706534000	0.900242000	2.596260000	1	2.580289000	1.385602000	-2.021551000
6	-0.798564000	-2.249753000	-0.180153000	6	-1.003529000	-1.507782000	-2.616738000
6	0.652378000	2.310893000	3.799718000	1	-0.051038000	-1.032710000	-2.336353000
1	0.710136000	3.353889000	4.150136000	6	-6.111004000	-1.318282000	-1.371814000
1	-0.303002000	2.172570000	3.270315000	1	-6.410976000	-1.361050000	-2.421451000
1	0.617520000	1.668298000	4.693223000	6	-0.713055000	-2.456963000	-3.778222000
6	2.760578000	-4.157113000	-0.851418000	1	0.010724000	-3.232462000	-3.485592000
1	2.734002000	-5.217691000	-1.116517000	1	-1.628533000	-2.964335000	-4.122653000
6	5.396930000	-0.126155000	0.045129000	1	-0.293971000	-1.907002000	-4.635583000
1	6.377258000	0.347508000	0.146901000	6	2.291811000	4.427359000	-0.576089000
6	5.218540000	-4.233872000	-0.888092000	1	2.412938000	5.069629000	-1.451087000
1	5.154509000	-5.296384000	-1.137428000	6	-1.964204000	-0.390168000	-3.023279000
6	-6.210409000	-2.212750000	0.872866000	1	-2.944667000	-0.792926000	-3.324657000
1	-6.588395000	-2.954526000	1.580151000	1	-2.128930000	0.309318000	-2.188753000
6	-3.405728000	3.120736000	-1.479462000	1	-1.560134000	0.183818000	-3.871870000
1	-4.220735000	2.464220000	-1.789805000	6	-6.602899000	-2.258898000	-0.465155000
6	2.039603000	4.193039000	1.806538000	1	-7.292691000	-3.035063000	-0.805604000
1	1.971640000	4.648279000	2.797771000	6	6.506989000	-2.264646000	-0.448370000

1	7.470009000	-1.757665000	-0.344650000
6	0.817961000	2.375339000	-2.703489000
1	0.123462000	1.858301000	-2.021290000
1	0.417452000	3.389981000	-2.853431000
1	0.800596000	1.848930000	-3.671118000
6	-3.216284000	1.881488000	0.672584000
6	-3.105256000	1.235724000	1.935600000

Method: PBE0/def2-SVP opt freq Empirical Dispersion=GD3BJ
imaginary frequencies: 0
Sum of electronic and zero-point Energies= –4116.596369 A.U.

TS1

31	0.214657000	0.429451000	0.340311000
6	3.259005000	0.696657000	-0.814292000
8	2.027168000	1.393562000	-2.920019000
7	-1.686119000	0.838522000	-0.148952000
6	-2.661104000	-1.391857000	0.102518000
7	-0.214194000	-1.504885000	0.068093000
6	1.635928000	-2.502004000	-1.177976000
6	-1.448193000	-3.549027000	0.185427000
1	-0.497940000	-4.080786000	0.152552000
6	-2.753972000	0.040108000	-0.057617000
6	4.348314000	-0.141456000	-0.402091000
6	1.361535000	5.041195000	0.812506000
1	0.730167000	5.868383000	0.480827000
6	-5.153563000	-1.507924000	0.202201000
6	-2.054812000	3.102163000	0.718921000
6	2.828590000	-3.229375000	-1.166157000
1	3.359207000	-3.403186000	-2.102744000
6	-4.067958000	0.635817000	-0.109915000
1	-4.124987000	1.714165000	-0.254322000
6	2.203138000	2.793578000	0.471726000
6	-1.852540000	2.237111000	-0.375604000
6	2.115845000	5.155159000	1.980350000
1	2.080611000	6.074283000	2.570204000
6	-1.407146000	-2.107750000	0.111072000
6	2.096481000	-1.752565000	-3.566897000
1	2.947899000	-1.160620000	-3.208247000
1	2.471126000	-2.710728000	-3.962044000
1	1.649493000	-1.196521000	-4.402769000
6	-6.294984000	-3.639556000	0.474799000
1	-7.219485000	-4.210866000	0.576917000
6	-1.716583000	2.729859000	-1.693421000
6	-1.464647000	1.789599000	-2.858887000
1	-0.814424000	0.983464000	-2.479386000
6	0.981532000	-2.285857000	0.055061000
6	-0.726614000	2.444033000	-4.020092000
1	-1.348599000	3.198333000	-4.528771000
1	0.216482000	2.899407000	-3.691070000
1	-0.464521000	1.679193000	-4.765690000
6	-2.609723000	-4.236654000	0.310185000
1	-2.593131000	-5.327527000	0.382139000
6	-5.201639000	-0.094006000	0.029456000
1	-6.176365000	0.400567000	0.002533000
6	-5.063694000	-4.283799000	0.471393000
1	-5.010714000	-5.371597000	0.565614000
6	6.271978000	-1.518560000	-0.945616000
1	6.959066000	-1.927998000	-1.690266000
6	1.400273000	3.868957000	0.064517000
1	0.797607000	3.773453000	-0.841551000
6	-1.827945000	4.109393000	-1.889437000
1	-1.724320000	4.515077000	-2.896742000
6	-3.878194000	-2.145286000	0.217425000
6	-3.421540000	3.024971000	2.842093000
1	-3.465669000	4.118630000	2.965013000
1	-4.312213000	2.712699000	2.276405000
1	-3.484188000	2.578767000	3.846742000
6	-2.052784000	4.975406000	-0.823017000
1	-2.135012000	6.050313000	-1.001542000
6	3.364623000	-3.719438000	0.019913000
1	4.306395000	-4.271639000	0.004763000
6	1.055820000	-1.963910000	-2.474418000
1	0.623345000	-0.974437000	-2.246313000
6	-0.079702000	-2.846891000	-2.997846000
1	0.292451000	-3.859520000	-3.222565000
1	-0.904059000	-2.946515000	-2.279492000
1	-0.494085000	-2.424323000	-3.926533000
6	4.525111000	-0.466584000	0.954257000
1	3.823169000	-0.072892000	1.691401000
6	5.236099000	-0.685813000	-1.349040000
1	5.103759000	-0.436770000	-2.404172000
6	2.718676000	-3.476488000	1.224473000
1	3.158708000	-3.843315000	2.155002000
6	-2.763864000	1.145835000	-3.349737000
1	-3.283953000	0.586866000	-2.560335000
1	-3.457275000	1.915401000	-3.726146000
1	-2.555203000	0.446292000	-4.174184000
6	2.917246000	4.089735000	2.392043000
1	3.514850000	4.174404000	3.303012000
6	2.965935000	2.918488000	1.640985000
1	3.604834000	2.091913000	1.956804000
6	-3.866474000	-3.565465000	0.342310000
6	1.521940000	-2.755791000	1.269412000
6	-2.135547000	2.586897000	2.143342000
1	-2.151231000	1.487412000	2.098608000
6	0.851185000	-2.484914000	2.603747000
1	-0.140266000	-2.054161000	2.399075000
6	5.557689000	-1.308748000	1.350639000
1	5.675447000	-1.560434000	2.407616000
6	0.625606000	-3.764563000	3.406931000
1	0.050471000	-4.502567000	2.828021000
1	1.576655000	-4.234852000	3.702234000
1	0.066102000	-3.546446000	4.329826000
6	-2.156247000	4.473522000	0.468108000
1	-2.309914000	5.159886000	1.304429000
6	1.635771000	-1.448456000	3.410282000
1	2.659521000	-1.798765000	3.618604000
1	1.711170000	-0.495729000	2.862163000
1	1.142882000	-1.246458000	4.374380000
6	6.437554000	-1.835125000	0.405055000
1	7.252673000	-2.491639000	0.718684000
6	-6.331066000	-2.257323000	0.334902000

1	-7.288398000	-1.729771000	0.321116000
6	-0.894777000	2.993188000	2.940347000
1	0.028595000	2.639658000	2.455193000
1	-0.815150000	4.088073000	3.027021000
1	-0.931958000	2.573143000	3.957952000
6	2.268985000	1.590175000	-0.369461000
6	2.422264000	1.248899000	-1.780448000

Method: PBE0/def2-SVP opt freq Empirical Dispersion=GD3BJ
imaginary frequencies: –174.7954
Sum of electronic and zero-point Energies= –4116.581139 A.U.

Int2

31	0.203597000	0.355609000	-0.058730000
6	2.879285000	0.182703000	-0.895846000
8	2.239492000	0.340520000	-3.311162000
7	-1.582071000	1.030802000	-0.196557000
6	-2.731781000	-1.138343000	-0.208788000
7	-0.311418000	-1.484237000	0.012244000
6	1.478596000	-2.968277000	-0.771628000
6	-1.764330000	-3.342008000	0.373725000
1	-0.881248000	-3.930123000	0.620379000
6	-2.690402000	0.293630000	-0.370832000
6	4.026063000	-0.514328000	-0.309659000
6	1.839588000	4.535568000	1.321178000
1	1.165483000	5.134799000	1.939516000
6	-5.198655000	-1.065767000	-0.589672000
6	-2.161238000	3.040720000	1.065051000
6	2.589823000	-3.755741000	-0.461988000
1	3.153679000	-4.227925000	-1.266952000
6	-3.909141000	0.981595000	-0.708955000
1	-3.848769000	2.056924000	-0.874161000
6	2.293333000	2.414587000	0.182800000
6	-1.670010000	2.456335000	-0.124873000
6	3.050480000	5.071611000	0.877605000
1	3.340384000	6.091190000	1.141223000
6	-1.567281000	-1.950996000	0.055902000
6	2.170426000	-2.935088000	-3.217044000
1	3.063778000	-2.371769000	-2.919463000
1	2.460298000	-3.991455000	-3.334597000
1	1.853564000	-2.566923000	-4.203288000
6	-6.550349000	-3.081421000	-0.412356000
1	-7.520961000	-3.577366000	-0.469294000
6	-1.181599000	3.234748000	-1.190376000
6	-0.675798000	2.610600000	-2.476105000
1	-0.393338000	1.567912000	-2.255864000
6	0.792918000	-2.356154000	0.298131000
6	0.578527000	3.293149000	-3.012139000
1	0.364290000	4.303383000	-3.395399000
1	1.347995000	3.374561000	-2.230536000
1	0.999306000	2.705013000	-3.840281000
6	-2.988201000	-3.925150000	0.340107000
1	-3.091402000	-4.988100000	0.573360000
6	-5.095805000	0.334940000	-0.826135000
1	-5.995915000	0.892131000	-1.098745000
6	-5.408274000	-3.801019000	-0.080437000
1	-5.474256000	-4.870892000	0.133566000
6	5.990037000	-1.937891000	-0.478093000
1	6.685585000	-2.510722000	-1.097184000
6	1.478101000	3.236630000	0.991361000
1	0.535743000	2.839189000	1.374848000
6	-1.203980000	4.625818000	-1.046296000
1	-0.820799000	5.251293000	-1.854145000
6	-4.011829000	-1.788406000	-0.273803000
6	-3.682463000	2.843414000	3.096505000
1	-3.284950000	3.703704000	3.657160000
1	-4.531397000	3.195016000	2.490992000
1	-4.066289000	2.126261000	3.837682000
6	-1.687115000	5.221032000	0.110151000
1	-1.686666000	6.309224000	0.205002000
6	3.012480000	-3.926762000	0.851972000
1	3.900337000	-4.525432000	1.064494000
6	1.034471000	-2.775920000	-2.211731000
1	0.661520000	-1.741060000	-2.297521000
6	-0.115617000	-3.711601000	-2.593520000
1	0.188675000	-4.763963000	-2.474347000
1	-1.013015000	-3.552853000	-1.982342000
1	-0.397086000	-3.558371000	-3.647005000
6	4.222883000	-0.455117000	1.076463000
1	3.527453000	0.139183000	1.671258000
6	4.940138000	-1.253428000	-1.079819000
1	4.828520000	-1.285409000	-2.167588000
6	2.324051000	-3.311426000	1.887497000
1	2.672260000	-3.435898000	2.915566000
6	-1.789262000	2.560026000	-3.524047000
1	-2.661622000	1.993008000	-3.164634000
1	-2.130940000	3.576004000	-3.779961000
1	-1.429063000	2.080189000	-4.447215000
6	3.886679000	4.270130000	0.099349000
1	4.844483000	4.663326000	-0.253113000
6	3.521216000	2.972279000	-0.242584000
1	4.187896000	2.367692000	-0.861538000
6	-4.152171000	-3.181949000	-0.010858000
6	1.203114000	-2.515359000	1.635615000
6	-2.615168000	2.180259000	2.232636000
1	-3.070922000	1.267690000	1.819661000
6	0.469586000	-1.863404000	2.794055000
1	-0.409298000	-1.339695000	2.386920000
6	5.277190000	-1.136746000	1.676285000
1	5.409857000	-1.076256000	2.759902000
6	-0.055452000	-2.904576000	3.782785000
1	-0.701946000	-3.642520000	3.284449000
1	0.768402000	-3.451947000	4.266976000
1	-0.643242000	-2.419478000	4.577704000
6	-2.161275000	4.433209000	1.155555000
1	-2.530366000	4.913830000	2.063017000
6	1.336299000	-0.815025000	3.491178000
1	2.255037000	-1.262945000	3.901152000
1	1.635474000	-0.022928000	2.788439000
1	0.787107000	-0.348315000	4.323556000
6	6.162322000	-1.891288000	0.906806000
1	6.989136000	-2.427393000	1.378749000
6	-6.437679000	-1.719831000	-0.663300000

1	-7.322655000	-1.131643000	-0.919522000
6	-1.421787000	1.743213000	3.084157000
1	-0.673579000	1.192912000	2.493362000
1	-0.910456000	2.613000000	3.524720000
1	-1.745800000	1.082368000	3.903354000
6	1.908364000	1.065470000	-0.195439000
6	2.566808000	0.255954000	-2.187576000

Method: PBE0/def2-SVP opt freq Empirical Dispersion=GD3BJ
imaginary frequencies: 0
Sum of electronic and zero-point Energies= –4116.598914 A.U.

TS2

31	0.226830000	0.288611000	-0.338723000
6	2.730016000	0.026602000	-0.956144000
8	1.750241000	-0.225438000	-3.243022000
7	-1.525534000	1.058223000	-0.416782000
6	-2.779581000	-1.038863000	-0.235354000
7	-0.390575000	-1.463706000	0.136181000
6	1.308948000	-3.185928000	-0.285638000
6	-1.950094000	-3.177170000	0.702734000
1	-1.113192000	-3.754902000	1.093824000
6	-2.668609000	0.366058000	-0.535401000
6	3.988877000	-0.647354000	-0.626269000
6	2.161958000	4.297165000	1.606358000
1	1.512553000	4.939977000	2.206364000
6	-5.224958000	-0.899536000	-0.715668000
6	-1.997892000	3.080958000	0.863284000
6	2.364200000	-3.958074000	0.208169000
1	2.891888000	-4.634337000	-0.466263000
6	-3.845876000	1.077483000	-0.955217000
1	-3.728621000	2.131032000	-1.209694000
6	2.475783000	2.216258000	0.364567000
6	-1.540638000	2.480744000	-0.332399000
6	3.461275000	4.703351000	1.294171000
1	3.843200000	5.662313000	1.652045000
6	-1.665259000	-1.859667000	0.191257000
6	2.064663000	-3.515473000	-2.681841000
1	2.887348000	-2.822058000	-2.466019000
1	2.460705000	-4.541278000	-2.612014000
1	1.748231000	-3.353263000	-3.722470000
6	-6.672031000	-2.828829000	-0.389702000
1	-7.660331000	-3.289284000	-0.440473000
6	-0.996726000	3.248551000	-1.381607000
6	-0.516527000	2.620103000	-2.675042000
1	-0.459577000	1.529615000	-2.525224000
6	0.662092000	-2.310598000	0.611413000
6	0.883794000	3.086753000	-3.064040000
1	0.894078000	4.148444000	-3.357191000
1	1.589090000	2.955915000	-2.230207000
1	1.253523000	2.496051000	-3.914752000
6	-3.196036000	-3.710738000	0.668201000
1	-3.364562000	-4.724494000	1.041071000
6	-5.054080000	0.469731000	-1.068991000
1	-5.918950000	1.034480000	-1.427044000
6	-5.580550000	-3.550057000	0.082697000
1	-5.707135000	-4.582166000	0.419792000
6	5.987254000	-1.857911000	-1.287215000
1	6.610700000	-2.290264000	-2.073960000
6	1.683713000	3.074467000	1.156117000
1	0.664879000	2.769614000	1.404220000
6	-0.933534000	4.635212000	-1.216707000
1	-0.510715000	5.247168000	-2.016279000
6	-4.085014000	-1.634895000	-0.277744000
6	-3.983897000	2.611720000	2.344134000
1	-4.089648000	3.659301000	2.667968000
1	-4.626074000	2.464155000	1.463543000
1	-4.366895000	1.972422000	3.154737000
6	-1.391501000	5.245413000	-0.055605000
1	-1.328788000	6.330523000	0.053346000
6	2.768732000	-3.864955000	1.534809000
1	3.603167000	-4.471487000	1.893571000
6	0.883630000	-3.302945000	-1.738547000
1	0.411181000	-2.347236000	-2.015629000
6	-0.151192000	-4.412140000	-1.944580000
1	0.260920000	-5.387578000	-1.638755000
1	-1.070524000	-4.240084000	-1.369611000
1	-0.428225000	-4.481286000	-3.008134000
6	4.394583000	-0.749049000	0.712288000
1	3.750320000	-0.329753000	1.487714000
6	4.804993000	-1.206300000	-1.623889000
1	4.505247000	-1.113520000	-2.671097000
6	2.128858000	-2.982337000	2.395809000
1	2.463168000	-2.901613000	3.433088000
6	-1.527768000	2.855543000	-3.798417000
1	-2.519420000	2.453725000	-3.539443000
1	-1.644696000	3.931532000	-4.005750000
1	-1.194059000	2.365404000	-4.725992000
6	4.261976000	3.872465000	0.508031000
1	5.276435000	4.183683000	0.244831000
6	3.777249000	2.657278000	0.037733000
1	4.404664000	2.033038000	-0.601989000
6	-4.302636000	-2.978648000	0.145216000
6	1.073921000	-2.180193000	1.952492000
6	-2.527279000	2.268868000	2.032938000
1	-2.494800000	1.206711000	1.752627000
6	0.401356000	-1.218469000	2.915262000
1	-0.337819000	-0.634644000	2.344443000
6	5.585027000	-1.386390000	1.044227000
1	5.887861000	-1.456135000	2.092125000
6	-0.366411000	-1.970196000	4.003396000
1	-1.112207000	-2.653822000	3.570345000
1	0.313593000	-2.568613000	4.630426000
1	-0.894917000	-1.263735000	4.662855000
6	-1.919249000	4.471089000	0.972080000
1	-2.265005000	4.955368000	1.888945000
6	1.395007000	-0.221815000	3.509521000
1	2.182481000	-0.728479000	4.089033000
1	1.875962000	0.369469000	2.715894000
1	0.881424000	0.474232000	4.190631000
6	6.383199000	-1.950572000	0.048174000
1	7.313390000	-2.460102000	0.311037000
6	-6.487985000	-1.508452000	-0.778501000

1	-7.334814000	-0.915550000	-1.133795000	1	-2.012317000	1.790208000	4.090677000
6	-1.642635000	2.425587000	3.270433000	6	1.955524000	0.942384000	-0.090104000
1	-0.602242000	2.136267000	3.062374000	6	1.973198000	-0.137180000	-2.072476000
1	-1.632581000	3.465671000	3.632582000				

Method: PBE0/def2-TZVPP opt freq Empirical Dispersion=GD3BJ
imaginary frequencies: –281.3142
Sum of electronic and zero-point Energies= –4116.589970 A.U.

2

31	0.350134000	0.146273000	-0.343630000	6	-4.176638000	-0.550334000	0.970105000
6	2.852638000	0.212109000	-0.463079000	6	-1.076182000	3.789619000	2.533754000
8	2.218978000	0.218769000	-2.817752000	1	-1.193059000	4.883865000	2.471746000
7	-1.179374000	1.330343000	-0.198181000	1	-2.049678000	3.332986000	2.303725000
6	-2.831978000	-0.347585000	0.503581000	1	-0.820769000	3.534601000	3.574381000
7	-0.729822000	-1.457744000	-0.111250000	6	-0.354460000	5.167650000	-1.725181000
6	0.042300000	-2.998217000	-1.838629000	1	-0.142514000	6.161978000	-2.125262000
6	-2.601113000	-2.816155000	0.465293000	6	1.388182000	-4.925966000	-1.227881000
1	-1.988015000	-3.685232000	0.230323000	1	1.934830000	-5.824138000	-1.524475000
6	-2.412271000	1.003402000	0.203270000	6	-0.660824000	-2.171937000	-2.900027000
6	4.306430000	0.066570000	-0.633018000	1	-0.724252000	-1.140832000	-2.516430000
6	1.797406000	0.723954000	4.390775000	6	-2.092529000	-2.664175000	-3.122887000
1	1.039588000	0.553924000	5.159446000	1	-2.092482000	-3.695637000	-3.510958000
6	-5.073026000	0.541207000	1.163960000	1	-2.681631000	-2.657475000	-2.194395000
6	-0.315405000	3.608329000	0.137618000	1	-2.610530000	-2.026190000	-3.856455000
6	0.750718000	-4.149717000	-2.189514000	6	5.046532000	-0.807730000	0.179294000
1	0.810387000	-4.440803000	-3.239428000	1	4.533324000	-1.367387000	0.964429000
6	-3.382797000	2.062953000	0.330439000	6	4.970430000	0.754532000	-1.660532000
1	-3.065608000	3.068304000	0.056070000	1	4.397334000	1.407147000	-2.319878000
6	2.463535000	0.634456000	2.042571000	6	1.345361000	-4.545011000	0.106699000
6	-0.909769000	2.641793000	-0.699236000	1	1.865943000	-5.145997000	0.856171000
6	3.045105000	1.242273000	4.737282000	6	-3.170342000	2.257110000	-3.461067000
1	3.273157000	1.478206000	5.779496000	1	-3.851095000	2.432429000	-2.614313000
6	-2.006010000	-1.514963000	0.276205000	1	-3.142029000	3.176682000	-4.067319000
6	0.110440000	-2.106038000	-4.213847000	1	-3.603018000	1.459775000	-4.085466000
1	1.129141000	-1.725217000	-4.055990000	6	3.996236000	1.469253000	3.740878000
1	0.164097000	-3.087094000	-4.711586000	1	4.970406000	1.889841000	4.002157000
1	-0.396595000	-1.421183000	-4.909173000	6	3.713176000	1.167783000	2.413377000
6	-6.830867000	-0.948649000	1.946772000	1	4.461830000	1.360641000	1.644092000
1	-7.841870000	-1.100284000	2.329225000	6	-4.687938000	-1.854966000	1.233472000
6	-1.189012000	2.912129000	-2.055136000	6	0.656012000	-3.397539000	0.511065000
6	-1.773209000	1.863061000	-2.981597000	6	0.019777000	3.304168000	1.583708000
1	-1.879975000	0.931726000	-2.406077000	1	0.069425000	2.209635000	1.677469000
6	-0.019579000	-2.646115000	-0.471383000	6	0.657361000	-3.003253000	1.974900000
6	-0.832882000	1.566278000	-4.149600000	1	0.182899000	-2.013165000	2.045064000
1	-0.670707000	2.460604000	-4.771969000	6	6.414530000	-0.968256000	-0.010219000
1	0.149209000	1.209505000	-3.802594000	1	6.974040000	-1.653999000	0.630872000
1	-1.267836000	0.790078000	-4.798383000	6	-0.159855000	-3.972251000	2.830960000
6	-3.857833000	-2.977005000	0.945478000	1	-1.205449000	-4.031430000	2.497254000
1	-4.256229000	-3.982470000	1.105855000	1	0.266033000	-4.987772000	2.793160000
6	-4.631665000	1.849145000	0.812114000	1	-0.159788000	-3.649440000	3.883915000
1	-5.322212000	2.688491000	0.930192000	6	-0.055670000	4.872352000	-0.399662000
6	-5.990118000	-2.032173000	1.721584000	1	0.396591000	5.638271000	0.233722000
1	-6.339308000	-3.049939000	1.914547000	6	2.080669000	-2.872854000	2.514400000
6	6.343023000	0.598583000	-1.841894000	1	2.592315000	-3.847767000	2.540958000
1	6.848041000	1.148089000	-2.640223000	1	2.676829000	-2.190817000	1.893023000
6	1.511718000	0.437317000	3.060117000	1	2.072312000	-2.470548000	3.538461000
1	0.524949000	0.054739000	2.785789000	6	7.069898000	-0.259227000	-1.018445000
6	-0.906454000	4.190147000	-2.544535000	1	8.145533000	-0.382991000	-1.166307000
1	-1.118530000	4.419391000	-3.591808000	6	-6.370012000	0.328691000	1.653652000

1	-7.021679000	1.195222000	1.793140000	1	1.662781000	3.465317000	2.986674000
6	1.382299000	3.852740000	1.995826000	6	2.079804000	0.362653000	0.661616000
1	2.165062000	3.551705000	1.284999000	6	1.911073000	0.171383000	-1.649338000
1	1.379885000	4.952688000	2.058366000				

Method: PBE0/def2-TZVPP opt freq Empirical Dispersion=GD3BJ
imaginary frequencies: 0
Sum of electronic and zero-point Energies= –4116.638269 A.U.

3

31	0.200425000	0.028131000	-0.467110000	6	1.748583000	-2.919471000	-2.829861000
8	0.604479000	0.432270000	-2.263448000	1	2.388224000	-2.448961000	-2.069214000
6	-2.290367000	1.387550000	0.009739000	1	1.906938000	-2.378003000	-3.774025000
7	-1.126622000	-1.346694000	-0.344992000	1	2.099574000	-3.954150000	-2.972076000
8	2.413489000	0.684432000	-3.536269000	6	0.270753000	-5.214582000	0.570178000
6	2.754332000	0.179918000	-1.217246000	1	0.647851000	-6.209267000	0.819034000
6	1.906813000	0.460036000	-2.461363000	6	-5.207276000	-0.748302000	-1.148402000
7	-0.966678000	1.407288000	0.184688000	6	6.364676000	-0.916242000	-0.977187000
6	4.223021000	0.155798000	-1.385332000	1	6.936076000	-1.745285000	-0.552671000
6	-2.426881000	-1.090541000	-0.572092000	6	-0.372384000	1.330726000	2.992422000
6	-1.703011000	-2.443562000	2.286796000	1	-0.333222000	0.436214000	2.347645000
1	-1.818193000	-1.407661000	1.934361000	6	1.187801000	3.788949000	-2.327217000
6	2.100795000	-0.084394000	-0.055964000	1	1.559886000	4.823020000	-2.245810000
6	2.373592000	-1.481338000	1.985280000	1	1.064765000	3.551940000	-3.393887000
1	1.645195000	-2.182808000	1.568347000	1	1.966092000	3.122089000	-1.930918000
6	-0.450085000	-4.486694000	1.510721000	6	3.658683000	0.552669000	1.799222000
1	-0.632728000	-4.916590000	2.498393000	1	3.928082000	1.450621000	1.239375000
6	-0.936071000	-3.208843000	1.222281000	6	-0.133403000	3.605684000	-1.578809000
6	0.038281000	-3.401309000	-1.031153000	1	-0.521658000	2.616550000	-1.859006000
6	-0.689054000	-2.675942000	-0.064924000	6	3.896141000	-0.852674000	3.753419000
6	-3.273285000	-2.192323000	-0.942713000	1	4.353827000	-1.052272000	4.725142000
1	-2.809087000	-3.175618000	-1.017476000	6	0.092488000	3.611473000	-0.079569000
6	2.723813000	-0.338789000	1.249356000	6	4.234502000	0.298453000	3.040504000
6	-4.420140000	0.357918000	-0.715305000	1	4.957431000	1.005473000	3.454827000
6	0.274840000	-2.868912000	-2.430199000	6	-6.572028000	-0.587351000	-1.434278000
1	-0.031508000	-1.813910000	-2.454050000	1	-7.138617000	-1.457288000	-1.776368000
6	7.019278000	0.114653000	-1.650111000	6	-0.600139000	-3.606583000	-3.445947000
1	8.107193000	0.100375000	-1.752641000	1	-0.352329000	-4.679890000	-3.481442000
6	-3.013731000	0.222082000	-0.456817000	1	-0.448704000	-3.190523000	-4.453790000
6	4.979788000	-0.893925000	-0.844328000	1	-1.669221000	-3.515333000	-3.201494000
1	4.470423000	-1.702855000	-0.316543000	6	-3.038166000	2.584686000	0.300497000
6	0.511730000	-4.671070000	-0.685397000	1	-2.490639000	3.429050000	0.717989000
1	1.077193000	-5.245179000	-1.422963000	6	0.010924000	2.515610000	2.124964000
6	4.889849000	1.175973000	-2.079244000	6	-1.158379000	4.654814000	-2.010186000
1	4.309683000	1.980522000	-2.531748000	1	-0.821956000	5.671888000	-1.751146000
6	2.964678000	-1.742239000	3.219486000	1	-2.136133000	4.490581000	-1.534414000
1	2.690402000	-2.646414000	3.768800000	1	-1.304993000	4.619182000	-3.100804000
6	-0.946213000	-2.387338000	3.612693000	6	-4.360365000	2.692731000	0.021329000
1	-0.834642000	-3.386349000	4.062209000	1	-4.883187000	3.633175000	0.214883000
1	-1.494496000	-1.766022000	4.337358000	6	-5.096368000	1.597092000	-0.518614000
1	0.057785000	-1.958415000	3.487558000	6	-7.201266000	0.640550000	-1.277713000
6	-3.104637000	-3.022766000	2.488516000	1	-8.263239000	0.752315000	-1.503702000
1	-3.681584000	-3.025199000	1.553100000	6	0.674116000	3.617909000	2.668456000
1	-3.663971000	-2.431317000	3.230212000	1	0.928096000	3.621365000	3.729445000
1	-3.051483000	-4.060372000	2.855631000	6	0.595055000	1.093621000	4.146083000
6	-4.583687000	-2.024203000	-1.253680000	1	0.536566000	1.893623000	4.900959000
1	-5.180447000	-2.877746000	-1.585917000	1	1.633779000	1.022122000	3.793861000
6	6.276594000	1.158622000	-2.200387000	1	0.347473000	0.152106000	4.656873000
1	6.781276000	1.965208000	-2.737858000	6	0.745652000	4.695051000	0.517586000
6	-0.303229000	2.542660000	0.748416000	1	1.052306000	5.538728000	-0.105290000

6	-6.462397000	1.720293000	-0.806438000	6	-1.808699000	1.460740000	3.504509000
1	-6.944072000	2.688111000	-0.644584000	1	-1.911296000	2.356003000	4.138467000
6	1.026053000	4.707131000	1.877430000	1	-2.085766000	0.583970000	4.110287000
1	1.540651000	5.561726000	2.323047000	1	-2.536835000	1.547636000	2.685871000

Method: PBE0/def2-TZVPP opt freq Empirical Dispersion=GD3BJ
imaginary frequencies: 0
Sum of electronic and zero-point Energies= –4191.808165 A.U.

4

31	-0.317470000	-0.020000000	0.612224000	6	0.099131000	-4.148104000	-2.439002000
16	-1.262427000	-0.079945000	2.631625000	1	1.193435000	-4.054075000	-2.387577000
8	-3.879296000	-0.105534000	2.643661000	1	-0.216686000	-3.851962000	-3.451540000
7	0.960484000	1.415558000	0.333402000	1	-0.155838000	-5.211710000	-2.304947000
7	0.981247000	-1.419667000	0.268166000	6	1.031976000	-3.029766000	2.098450000
6	3.838137000	0.061993000	-1.610484000	6	-1.801986000	0.019993000	-4.724550000
6	-0.164658000	3.598497000	0.092388000	1	-1.727606000	0.027398000	-5.814607000
6	2.103644000	1.297684000	-0.350112000	6	6.164725000	0.117582000	-3.265636000
6	2.645855000	0.034737000	-0.806264000	1	7.047149000	0.139049000	-3.907668000
6	-2.010252000	-0.010076000	-0.422837000	6	0.887779000	2.959437000	2.221868000
6	4.462318000	1.288765000	-1.980364000	6	1.861128000	-2.066469000	2.925051000
6	0.565463000	2.681852000	0.875593000	1	1.706564000	-1.064204000	2.496757000
6	2.116728000	-1.255803000	-0.418472000	6	-0.227170000	5.104680000	1.996971000
6	-2.008577000	0.003077000	-1.895735000	1	-0.537494000	6.055176000	2.437217000
6	4.469739000	-1.137304000	-2.051312000	6	0.481405000	4.182387000	2.759434000
6	2.856114000	-2.435502000	-0.794853000	1	0.719526000	4.415147000	3.798765000
1	2.479706000	-3.394807000	-0.442748000	6	0.169686000	4.276290000	-2.317471000
6	3.932793000	2.501456000	-1.452379000	1	-0.081816000	4.016309000	-3.357581000
1	4.432067000	3.443933000	-1.692730000	1	1.263335000	4.230291000	-2.214484000
6	-3.079035000	-0.023518000	0.415769000	1	-0.142686000	5.319273000	-2.148418000
6	-3.154798000	-0.039818000	-2.715021000	6	-5.233832000	-1.194299000	-0.105896000
1	-4.143663000	-0.083835000	-2.264974000	1	-4.735958000	-2.144449000	0.100168000
6	2.838043000	2.501265000	-0.653788000	6	-3.049381000	-0.031016000	-4.102204000
1	2.456357000	3.436038000	-0.245933000	1	-3.959736000	-0.065420000	-4.705624000
6	0.627565000	-2.708205000	0.784846000	6	-2.113790000	-3.366725000	-1.531320000
6	-0.140282000	-3.603161000	0.013731000	1	-2.469753000	-4.402214000	-1.409965000
6	-0.471253000	-4.840733000	0.575062000	1	-2.429927000	-3.015123000	-2.524941000
1	-1.061426000	-5.549313000	-0.011210000	1	-2.616294000	-2.742668000	-0.779687000
6	3.947654000	-2.382348000	-1.596108000	6	1.686842000	1.978103000	3.056882000
1	4.449856000	-3.306160000	-1.895497000	1	1.515990000	0.980006000	2.624883000
6	-0.533060000	3.322226000	-1.351009000	6	-5.168647000	1.213160000	-0.210526000
1	-0.194611000	2.305760000	-1.590587000	1	-4.619346000	2.149301000	-0.088829000
6	-0.762325000	0.047110000	-2.552641000	6	-2.045524000	3.355670000	-1.559867000
1	0.156352000	0.071395000	-1.961986000	1	-2.450532000	4.370048000	-1.415679000
6	-0.652128000	0.057577000	-3.938523000	1	-2.553894000	2.682196000	-0.857104000
1	0.336866000	0.093258000	-4.401242000	1	-2.300622000	3.030156000	-2.579777000
6	5.604918000	-1.092152000	-2.873409000	6	1.421716000	-2.006784000	4.384314000
1	6.052593000	-2.036377000	-3.194312000	1	0.349646000	-1.775574000	4.464595000
6	-0.547132000	4.809423000	0.678531000	1	1.982387000	-1.222482000	4.914992000
1	-1.110531000	5.532883000	0.084051000	1	1.615999000	-2.953321000	4.912566000
6	-0.066111000	-5.181552000	1.858903000	6	-7.207209000	0.037794000	-0.757351000
1	-0.336288000	-6.152966000	2.279532000	1	-8.254775000	0.052558000	-1.067544000
6	-2.917402000	-0.070949000	1.920904000	6	-6.569310000	-1.175351000	-0.500748000
6	-4.514849000	-0.001128000	0.023464000	1	-7.117076000	-2.115154000	-0.604595000
6	0.675519000	-4.278084000	2.612424000	6	-6.505001000	1.232325000	-0.603248000
1	0.979813000	-4.545768000	3.626123000	1	-7.001806000	2.187931000	-0.787852000
6	-0.595727000	-3.275131000	-1.393245000	6	3.353580000	-2.382792000	2.807745000
1	-0.312610000	-2.234209000	-1.597463000	1	3.573063000	-3.383847000	3.212990000
6	5.598113000	1.298839000	-2.802754000	1	3.950793000	-1.649744000	3.372945000
1	6.040579000	2.262901000	-3.066871000	1	3.695529000	-2.363198000	1.762653000

6	1.232182000	1.922613000	4.511105000	6	3.187086000	2.262384000	2.957047000
1	1.455864000	2.856937000	5.049544000	1	3.542481000	2.236582000	1.916689000
1	1.758704000	1.114063000	5.039750000	1	3.761337000	1.515898000	3.528543000
1	0.152279000	1.727315000	4.579552000	1	3.422964000	3.257999000	3.366316000

Method: PBE0/def2-TZVPP opt freq Empirical Dispersion=GD3BJ
imaginary frequencies: 0
Sum of electronic and zero-point Energies= –4514.649244 A.U.
